# Supplementary material for: Human cerebellum and ventral tegmental area interact during extinction of learned fear
Source: eLife. 2026 Jul 13;14:RP105399. doi: 10.7554/eLife.105399 (PMC13363218; doi:10.7554/eLife.105399)
Supplement: Supplementary file 1. — Results are shown separately for habituation, fear acquisition training, extinction training, recall, reacquisition, reextinction, and the unexpected unconditioned stimulus (US) phase. Factors included Stimulus (CS+ vs. CS-), Time (early vs. late halves of each phase), and the Stimulus × Time interaction. Reported statistics include numerator degrees of freedom, F-values, and p-values. Significance levels are indicated as *p<0.05; **p<0.01; ***p<0.001. [file elife-105399-supp1.docx]

# Supplementary information

## Skin conductance responses

### Non-parametric ANOVA SCR results

**Supplementary file 1:** Non-parametric ANOVA-type statistics for skin conductance responses (SCRs). Results are shown separately for habituation, fear acquisition training, extinction training, recall, reacquisition, reextinction, and the unexpected US phase. Factors included Stimulus (CS+ vs. CS-), Time (early vs. late halves of each phase), and the Stimulus x Time interaction. Reported statistics include numerator degrees of freedom, F-values, and p-values. Significance levels are indicated as * p < 0.05; ** p < 0.01; *** p < 0.001.

| **Factor** |  | **Numerator Df** | ***F*** | ***p*** |
| --- | --- | --- | --- | --- |
| *Habituation* | | | | |
| Stimulus |  | 1 | 1.19 | 0.276 |
| Time |  | 1 | 26.49 | **<0.001***** |
| Stimulus x Time |  | 1 | 0.06 | 0.802 |
| *Fear acquisition training* | | | | |
| Stimulus |  | 1 | 20.79 | **<0.001***** |
| Time |  | 1 | 28.75 | **<0.001***** |
| Stimulus x Time |  | 1 | 3.80 | 0.051 |
| *Extinction training* | | | | |
| Stimulus |  | 1 | 7.71 | **0.006**** |
| Time |  | 1 | 25.05 | **<0.001***** |
| Stimulus x Time |  | 1 | 3.74 | 0.053 |
| *Recall* | | | | |
| Stimulus |  | 1 | 4.99 | **0.026*** |
| Time |  | 1 | 36.74 | **<0.001***** |
| Stimulus x Time |  | 1 | 5.53 | **0.019*** |
| *Reacquisition* | | | | |
| Stimulus |  | 1 | 27.20 | **<0.001***** |
| Time |  | 1 | 35.44 | **<0.001***** |
| Stimulus x Time |  | 1 | 6.80 | **0.009**** |
| *Reextinction* | | | | |
| Stimulus |  | 1 | 4.47 | **0.035*** |
| Time |  | 1 | 0.08 | 0.780 |
| Stimulus x Time |  | 1 | 0.95 | 0.329 |
| *Unexpected US phase* | | | | |
| Stimulus |  | 1 | 5.43 | **0.020*** |
| Time |  | 1 | 0.29 | 0.593 |
| Stimulus x Time |  | 1 | 2.34 | 0.126 |
