# Supplementary material for: Human cerebellum and ventral tegmental area interact during extinction of learned fear
Source: eLife. 2026 Jul 13;14:RP105399. doi: 10.7554/eLife.105399 (PMC13363218; doi:10.7554/eLife.105399)
Supplement: Supplementary file 2. — Results are shown separately for habituation, fear acquisition training, extinction training, recall, reacquisition, reextinction, and the unexpected unconditioned stimulus (US) phase. Factors included Stimulus (CS+ vs. CS-), Time (first three vs. last three trials), and the Stimulus × Time interaction. Significance levels are indicated as *p<0.05; **p<0.01; ***p<0.001. [file elife-105399-supp2.docx]

# Supplementary information

## Skin conductance responses

### Non-parametric ANOVA SCR results (first-three and last-three trial analysis)

**Supplementary file 2:** Non-parametric ANOVA-type statistics for skin conductance responses (SCRs) based on the first-three and last-three trial analysis. Results are shown separately for habituation, fear acquisition training, extinction training, recall, reacquisition, reextinction, and the unexpected US phase. Factors included Stimulus (CS+ vs. CS-), Time (first three vs. last three trials), and the Stimulus x Time interaction. Significance levels are indicated as * p < 0.05; ** p < 0.01; *** p < 0.001.

| **Factor** | **Numerator Df** | ***F*** | ***p*** |
| --- | --- | --- | --- |
| *Habituation* | | | |
| Stimulus | 1 | 1.19 | 0.276 |
| Time | 1 | 26.49 | **<0.001***** |
| Stimulus x Time | 1 | 0.06 | 0.802 |
| *Fear acquisition training* | | | |
| Stimulus | 1 | 8.22 | **0.004**** |
| Time | 1 | 40.06 | **<0.001***** |
| Stimulus x Time | 1 | 4.05 | **0.044*** |
| *Extinction training* | | | |
| Stimulus | 1 | 4.99 | **0.026*** |
| Time | 1 | 34.62 | **<0.001***** |
| Stimulus x Time | 1 | 3.36 | 0.067 |
| *Recall* | | | |
| Stimulus | 1 | 4.24 | **0.039*** |
| Time | 1 | 47.42 | **<0.001***** |
| Stimulus x Time | 1 | 6.35 | **0.012*** |
| *Reacquisition* | | | |
| Stimulus | 1 | 20.47 | **<0.001***** |
| Time | 1 | 50.62 | **<0.001***** |
| Stimulus x Time | 1 | 8.70 | **0.003**** |
| *Reextinction* | | | |
| Stimulus | 1 | 9.36 | **0.002**** |
| Time | 1 | 0.02 | 0.895 |
| Stimulus x Time | 1 | 0.47 | 0.494 |
| *Unexpected US phase* | | | |
| Stimulus | 1 | 4.02 | **0.045*** |
| Time | 1 | 0.83 | 0.363 |
| Stimulus x Time | 1 | 4.67 | **0.031*** |
