# Supplementary material for: Human cerebellum and ventral tegmental area interact during extinction of learned fear
Source: eLife. 2026 Jul 13;14:RP105399. doi: 10.7554/eLife.105399 (PMC13363218; doi:10.7554/eLife.105399)
Supplement: Supplementary file 3. — Results are shown separately for habituation, fear acquisition training, extinction training, recall, reacquisition, reextinction, and the unexpected unconditioned stimulus (US) phase. Factors included Stimulus (CS+ vs. CS-), Time (early vs. late halves of each phase), and the Stimulus × Time interaction. Significance levels are indicated as *p<0.05; **p<0.01; ***p<0.001. [file elife-105399-supp3.docx]

# Supplementary information

## Pupil size responses

### Non-parametric ANOVA PSR results

**Supplementary file 3:** Non-parametric ANOVA-type statistics for pupil size responses (PSRs). Results are shown separately for habituation, fear acquisition training, extinction training, recall, reacquisition, reextinction, and the unexpected US phase. Factors included Stimulus (CS+ vs. CS-), Time (early vs. late halves of each phase), and the Stimulus x Time interaction. Significance levels are indicated as * p < 0.05; ** p < 0.01; *** p < 0.001.

| **Factor** | **Numerator Df** | ***F*** | ***p*** |
| --- | --- | --- | --- |
| *Habituation* | | | |
| Stimulus | 1 | 1.65 | 0.200 |
| Time | 1 | 0.26 | 0.609 |
| Stimulus x Time | 1 | 0.04 | 0.849 |
| *Fear acquisition training* | | | |
| Stimulus | 1 | 39.51 | **<0.001***** |
| Time | 1 | 24.31 | **<0.001***** |
| Stimulus x Time | 1 | 0.58 | 0.446 |
| *Extinction training* | | | |
| Stimulus | 1 | 1.12 | 0.290 |
| Time | 1 | 18.21 | **<0.001***** |
| Stimulus x Time | 1 | 4.28 | **0.038*** |
| *Recall* | | | |
| Stimulus | 1 | 6.59 | **0.010*** |
| Time | 1 | 6.41 | **0.011*** |
| Stimulus x Time | 1 | 4.87 | **0.027*** |
| *Reacquisition* | | | |
| Stimulus | 1 | 47.87 | **<0.001***** |
| Time | 1 | 4.54 | **0.033*** |
| Stimulus x Time | 1 | 7.70 | **0.006*** |
| *Reextinction* | | | |
| Stimulus | 1 | 9.10 | **0.003*** |
| Time | 1 | 0.83 | 0.364 |
| Stimulus x Time | 1 | 4.43 | **0.035*** |
| *Unexpected US phase* | | | |
| Stimulus | 1 | 17.35 | **<0.001***** |
| Time | 1 | 0.83 | 0.363 |
| Stimulus x Time | 1 | 0.03 | 0.872 |
