# Supplementary material for: Human cerebellum and ventral tegmental area interact during extinction of learned fear
Source: eLife. 2026 Jul 13;14:RP105399. doi: 10.7554/eLife.105399 (PMC13363218; doi:10.7554/eLife.105399)
Supplement: Supplementary file 4. — Results are shown separately for habituation, fear acquisition training, extinction training, recall, reacquisition, reextinction, and the unexpected unconditioned stimulus (US) phase. Factors included Stimulus (CS+ vs. CS-), Time (first three vs. last three trials), and the Stimulus × Time interaction. Significance levels are indicated as *p<0.05; **p<0.01; ***p<0.001. [file elife-105399-supp4.docx]

# Supplementary information

## Pupil size responses

### Non-parametric ANOVA PSR results (first-three and last-three trial analysis)

**Supplementary file 4:** Non-parametric ANOVA-type statistics for pupil size responses (PSRs) based on the first three and last three trial analysis. Results are shown separately for habituation, fear acquisition training, extinction training, recall, reacquisition, reextinction, and the unexpected US phase. Factors included Stimulus (CS+ vs. CS-), Time (first three vs. last three trials), and the Stimulus x Time interaction. Significance levels are indicated as * p < 0.05; ** p < 0.01; *** p < 0.001.

| **Factor** | **Numerator Df** | ***F*** | ***p*** |
| --- | --- | --- | --- |
| *Habituation* | | | |
| Stimulus | 1 | 1.65 | 0.200 |
| Time | 1 | 0.26 | 0.609 |
| Stimulus x Time | 1 | 0.04 | 0.849 |
| *Fear acquisition training* | | | |
| Stimulus | 1 | 29.02 | **<0.001***** |
| Time | 1 | 30.93 | **<0.001***** |
| Stimulus x Time | 1 | 0.35 | 0.554 |
| *Extinction training* | | | |
| Stimulus | 1 | 0.93 | 0.334 |
| Time | 1 | 41.57 | **<0.001***** |
| Stimulus x Time | 1 | 7.72 | **0.006**** |
| *Recall* | | | |
| Stimulus | 1 | 4.72 | **0.030*** |
| Time | 1 | 12.23 | **<0.001***** |
| Stimulus x Time | 1 | 7.13 | **0.008**** |
| *Reacquisition* | | | |
| Stimulus | 1 | 39.99 | **<0.001***** |
| Time | 1 | 9.40 | **0.002**** |
| Stimulus x Time | 1 | 8.22 | **0.004**** |
| *Reextinction* | | | |
| Stimulus | 1 | 4.44 | **0.035*** |
| Time | 1 | 0.36 | 0.547 |
| Stimulus x Time | 1 | 2.63 | 0.105 |
| *Unexpected US phase* | | | |
| Stimulus | 1 | 4.36 | **0.037*** |
| Time | 1 | 0.16 | 0.689 |
| Stimulus x Time | 1 | 0.14 | 0.705 |
