# Supplementary material for: Human cerebellum and ventral tegmental area interact during extinction of learned fear
Source: eLife. 2026 Jul 13;14:RP105399. doi: 10.7554/eLife.105399 (PMC13363218; doi:10.7554/eLife.105399)
Supplement: Supplementary file 5. — Median values and interquartile ranges (in parentheses) are shown for arousal, fear, unconditioned stimulus (US) expectancy, and valence ratings for conditioned stimulus (CS+) and CS- assessed after habituation, acquisition training, extinction training, recall test, and at the end of day 3. Rating scales ranged from 1 to 9, with anchors as indicated for each measure. Interquartile ranges are shown in parentheses. Statistically significant differences between CS+ and CS- are indicated in bold (least squares means tests; p<0.01). [file elife-105399-supp5.docx]

## Self-reports

### Self-reports results summary

***Supplementary file 5:*** *Summary of self-report ratings. Median values and interquartile ranges (in parentheses) are shown for arousal, fear, US expectancy, and valence ratings for CS+ and CS- assessed after habituation, acquisition training, extinction training, recall test, and at the end of day 3. Rating scales ranged from 1 to 9, with anchors as indicated for each measure. Interquartile ranges are shown in parentheses. Statistically significant differences between CS+ and CS- are indicated in bold (least squares means tests; p < 0.01).*

| Stimulus | Time of assessment | | | | |
| --- | --- | --- | --- | --- | --- |
|  | Post habituation | Post acquisition | Post extinction | Post recall | End of day 3 |
| *Arousal ratings (1 - very calm, 9 - very excited)* | | | | | |
| CS+ | 2 (1-3) | **6 (5-7)** | **3 (1-6)** | **3 (1-6)** | **7 (4-7)** |
| CS- | 2 (1-4) | **2 (1-3)** | **1 (1-3)** | **1 (1-3)** | **1 (1-2)** |
| *Fear ratings (1 - not afraid, 9 - very afraid)* | | | | | |
| CS+ | 1 (1-2) | **6 (3-6)** | **2 (1-5)** | **2 (1-4)** | **5 (3-7)** |
| CS- | 1 (1-2) | **1 (1-2)** | **1 (1-2)** | **1 (1-2)** | **1 (1-2)** |
| *US expectancy (1 - US not expected, 5 - unsure, 9 - US expected)* | | | | | |
| CS+ | 1 (1-2) | **9 (9-9)** | **3 (1-5)** | **3 (1-5)** | **6 (5-7)** |
| CS- | 1 (1-2) | **1 (1-2)** | **1 (1-1)** | **1 (1-2)** | **1 (1-2)** |
| *Valence ratings (1 - unpleasant, 9 - very pleasant)* | | | | | |
| CS+ | 7 (5-8) | **3 (2-4)** | **5 (4-7)** | **6 (5-8)** | **3 (3-5)** |
| CS- | 7 (5-9) | **8 (6-9)** | **7 (5-9)** | **8 (6-9)** | **8 (6-9)** |
