# Supplementary material for: Human cerebellum and ventral tegmental area interact during extinction of learned fear
Source: eLife. 2026 Jul 13;14:RP105399. doi: 10.7554/eLife.105399 (PMC13363218; doi:10.7554/eLife.105399)
Supplement: Supplementary file 6. — Results are shown for arousal, fear, valence, and unconditioned stimulus (US) expectancy ratings, with Stimulus (CS+ vs. CS-) and Time of assessment as within-subject factors, as well as the Stimulus × Time interaction. Degrees of freedom, F-values, and p-values are reported for each effect. Significance levels are indicated as *p<0.05; **p<0.01; ***p<0.001. [file elife-105399-supp6.docx]

## Self-reports

### Non-parametric ANOVA results for self-reports

***Supplementary file 6:*** *Non-parametric ANOVA-type statistics for self-report measures. Results are shown for arousal, fear, valence, and US expectancy ratings, with Stimulus (CS+ vs. CS-) and Time of assessment as within-subject factors, as well as the Stimulus x Time interaction. Degrees of freedom, F values, and p values are reported for each effect. Significance levels are indicated as * p < 0.05; ** p < 0.01; *** p < 0.001.*

| **Factor** | **Df** | **F** | **p** |
| --- | --- | --- | --- |
| *Arousal* | | | |
| Stimulus | 1 | 128.71 | **<0.001***** |
| Time | 3.51 | 16.31 | **<0.001***** |
| Stimulus x Time | 3.29 | 35.06 | **<0.001***** |
| *Fear* | | | |
| Stimulus | 1 | 105.13 | **<0.001***** |
| Time | 3.32 | 18.60 | **<0.001***** |
| Stimulus x Time | 3.4 | 34.47 | **<0.001***** |
| *Valence* | | | |
| Stimulus | 1 | 124.79 | **<0.001***** |
| Time | 3.39 | 19.80 | **<0.001***** |
| Stimulus x Time | 3.11 | 51.42 | **<0.001***** |
| *US expectancy* | | | |
| Stimulus | 1 | 451.62 | **<0.001***** |
| Time | 2.4 | 62.78 | **<0.001***** |
| Stimulus x Time | 2.8 | 187.33 | **<0.001***** |
