# Supplementary material for: Human cerebellum and ventral tegmental area interact during extinction of learned fear
Source: eLife. 2026 Jul 13;14:RP105399. doi: 10.7554/eLife.105399 (PMC13363218; doi:10.7554/eLife.105399)
Supplement: Supplementary file 7. — For each contrast and region of interest (CB: cerebellar cortex; DCN: deep cerebellar nuclei; VTA: ventral tegmental area), the table reports mean contrast estimates, 95% confidence intervals (CI), and Cohen’s d (one-sample effect size relative to zero). Effect sizes with Cohen’s d>0.5 and 95% confidence intervals entirely above zero are highlighted in bold to facilitate interpretation of effect magnitude and consistency across participants. Event-based contrasts show comparatively consistent effects despite being based on a small number of trials (e.g. first three unexpected US omissions), whereas parametric modulation and psychophysiological interaction (PPI) analyses incorporate a larger number of observations and show greater inter-individual variability. [file elife-105399-supp7.docx]

## Supplementary fMRI results

### Summary statistics for VOI contrast estimates across fMRI contrasts

***Supplementary file 7:*** *Summary statistics for subject-level VOI contrast estimates across fMRI contrasts. For each contrast and region of interest (CB: cerebellar cortex; DCN: deep cerebellar nuclei; VTA: ventral tegmental area), the table reports mean contrast estimates, 95% confidence intervals (CI), and Cohen’s d (one-sample effect size relative to zero). Effect sizes with Cohen’s d > 0.5 and 95% confidence intervals entirely above zero are highlighted in bold to facilitate interpretation of effect magnitude and consistency across participants. Event-based contrasts show comparatively consistent effects despite being based on a small number of trials (e.g., first three unexpected US omissions), whereas parametric modulation and psychophysiological interaction (PPI) analyses incorporate a larger number of observations and show greater inter-individual variability.*

| **Contrast** | **ROI** | **Mean** | **95% CI** | **Cohen’s d** |
| --- | --- | --- | --- | --- |
| *Figure 4: Acquisition, US prediction and presentation* | | | | |
| **A:** CS+ > CS- | CB | 0.173 | [-0.373, 0.720] | 0.097 |
|  | DCN | -0.200 | [-0.773, 0.373] | -0.107 |
|  | VTA | 0.888 | **[0.458, 1.319]** | **0.635** |
| **B:** CS+ x P (inv) | CB | 1.872 | **[0.894, 2.849]** | **0.589** |
|  | DCN | 0.562 | [-0.406, 1.529] | 0.179 |
|  | VTA | 2.078 | **[1.281, 2.874]** | **0.803** |
| **C:** US presentation | CB | 5.367 | **[3.879, 6.855]** | **1.110** |
|  | DCN | 2.040 | **[1.131, 2.950]** | **0.691** |
|  | VTA | 2.852 | **[2.064, 3.639]** | **1.115** |
| *Figure 5: Extinction, US prediction and omission* | | | | |
| **A:** CS+ > CS- | CB | -0.057 | [-0.566, 0.452] | -0.034 |
|  | DCN | -0.143 | [-0.649, 0.363] | -0.087 |
|  | VTA | 0.223 | [-0.170, 0.615] | 0.175 |
| **B:** CS+ x Prediction | CB | 1.043 | [-0.083, 2.169] | 0.285 |
|  | DCN | 0.356 | [-0.468, 1.180] | 0.133 |
|  | VTA | 2.057 | **[1.248, 2.865]** | **0.783** |
| **C:** US presentation | CB | 0.471 | [-0.062, 1.004] | 0.272 |
|  | DCN | 0.311 | [-0.208, 0.829] | 0.184 |
|  | VTA | 0.120 | [-0.351, 0.590] | 0.078 |
| *Figure 6: Unexpected US omission (event-based)* | | | | |
| **A:** Extinction | CB | 4.487 | **[2.762, 6.211]** | **0.801** |
|  | DCN | 1.989 | **[0.653, 3.325]** | 0.458 |
|  | VTA | 1.807 | **[0.616, 2.997]** | 0.467 |
| **B:** Recall | CB | 4.485 | **[2.823, 6.148]** | **0.830** |
|  | DCN | 1.352 | **[0.072, 2.632]** | 0.325 |
|  | VTA | 1.949 | **[0.903, 2.996]** | **0.573** |
| **C:** Reacquisition | CB | 8.082 | **[6.209, 9.955]** | **1.328** |
|  | DCN | 0.872 | [-0.379, 2.123] | 0.215 |
|  | VTA | 3.711 | **[2.133, 5.289]** | **0.724** |
| **D:** Reextinction | CB | 5.780 | **[3.748, 7.811]** | **0.876** |
|  | DCN | 0.969 | [-0.424, 2.361] | 0.214 |
|  | VTA | 3.152 | **[1.699, 4.605]** | **0.668** |
| *Figure 7: Unexpected US omission (parametric modulation)* | | | | |
| **A:** Extinction | CB | 3.409 | **[2.034, 4.784]** | **0.763** |
|  | DCN | 1.114 | **[0.259, 1.970]** | 0.401 |
|  | VTA | 1.871 | **[0.966, 2.777]** | **0.636** |
| **B:** Recall | CB | 4.029 | **[2.818, 5.241]** | **1.024** |
|  | DCN | 1.360 | [-0.217, 2.938] | 0.265 |
|  | VTA | 1.786 | **[0.696, 2.877]** | **0.504** |
| **C:** Reacquisition | CB | 2.675 | **[1.221, 4.128]** | **0.566** |
|  | DCN | 0.192 | [-0.916, 1.301] | 0.053 |
|  | VTA | 1.712 | **[0.398, 3.025]** | 0.401 |
| **D:** Reextinction | CB | 1.505 | [-0.742, 3.753] | 0.206 |
|  | DCN | 0.671 | [-1.381, 2.724] | 0.101 |
|  | VTA | 1.256 | [-0.551, 3.063] | 0.214 |
| *Figure 8: Unexpected US omission (PPI, VTA seed)* | | | | |
| **A:** Extinction | CB | -2.069 | [-6.848, 2.710] | -0.133 |
| **A:** Recall | CB | 3.209 | [-1.952, 8.370] | 0.191 |
| **A:** Reacquisition | CB | 2.558 | [-0.613, 5.729] | 0.248 |
| **A:** Reextinction | CB | 2.585 | [-1.824, 6.994] | 0.180 |
| **B:** All | CB | 1.571 | [-0.593, 3.735] | 0.223 |
