# Supplementary material for: Human cerebellum and ventral tegmental area interact during extinction of learned fear
Source: eLife. 2026 Jul 13;14:RP105399. doi: 10.7554/eLife.105399 (PMC13363218; doi:10.7554/eLife.105399)
Supplement: Supplementary file 8. — Clusters were identified in the cerebellar cortex, deep cerebellar nuclei (DCN), and ventral tegmental area (VTA) using threshold-free cluster enhancement (TFCE) with family-wise error (FWE) correction (p<0.05). Up to three local maxima per cluster are reported, separated by at least 8 mm. Coordinates are given in MNI space (x, y, z). Cluster size is reported as number of voxels (voxel volume = 3.375 mm³). US: unconditioned stimulus; CS: conditioned stimulus; VTA: ventral tegmental area; DCN: deep cerebellar nuclei; DN: dentate nucleus; IN: interposed nucleus; FN: fastigial nucleus; MNI: Montreal Neurological Institute standard brain; TFCE t: threshold-free cluster-enhanced t-statistic; pFWE: family-wise error-corrected p-value. [file elife-105399-supp8.docx]

## Supplementary fMRI results

### fMRI activation cluster tables

#### *fMRI activations related to the prediction and presentation of the US. TFCE and FWE corrected.*

***Supplementary file 8:*** *fMRI activation clusters related to the prediction, presentation and omission of the unconditioned stimulus (US) during acquisition and extinction training (Figure 4 and 5). Clusters were identified in the cerebellar cortex, deep cerebellar nuclei (DCN), and ventral tegmental area (VTA) using threshold-free cluster enhancement (TFCE) with family-wise error (FWE) correction (p < 0.05). Up to three local maxima per cluster are reported, separated by at least 8 mm. Coordinates are given in MNI space (x, y, z). Cluster size is reported as number of voxels (voxel volume = 3.375 mm³).*
*US: unconditioned stimulus; CS: conditioned stimulus; VTA: ventral tegmental area; DCN: deep cerebellar nuclei; DN: dentate nucleus; IN: interposed nucleus; FN: fastigial nucleus; MNI: Montreal Neurological Institute standard brain; TFCE t: threshold-free cluster-enhanced t-statistic; pFWE: family-wise error-corrected p-value.*

| **Index** | **Location (lobule, DCN, VTA)** | **Side** | **MNI coordinates/mm** | | | **Cluster size (number of voxels)** | **p_FWE_** | **TFCE t** |
| --- | --- | --- | --- | --- | --- | --- | --- | --- |
|  |  |  | **x** | **y** | **z** |  |  |  |
| *Figure 4A: Acquisition, CS+ > CS-, t-test, TFCE, p < 0.05, FWE corr.* | | | | | | | | |
| 1 | VTA | right | 4.0 | -16.0 | -13.0 | 27 | 0.016 | 1599 |
| 2 | VTA | left | -6.5 | -17.5 | -11.5 | 14 | 0.021 | 1503 |
| 3 | VTA | left | -2.0 | -17.5 | -10.0 | 1 | 0.036 | 1293 |
| *Figure 4B: CS+ x prediction (inverted) during acquisition, t-test, TFCE, p < 0.05, FWE corr.* | | | | | | | | |
| 1 | Extended cluster | left VI (2109), right VI (1758), right V (1324), left Crus I (1315), white matter (802), left V (748), right I-IV (643), left Crus II (506), right Crus I (453), vermal VI (380), left I-IV (366), left VIIb (137), left VIIIa (90), vermal VIIIa (57), vermal IX (53), left IX (46), right DN (40), vermal X (38), right IX (35), vermal VIIIb (33), vermal VIIb (23), right VIIIa (15), right X (7), right VIIb (5), right FN (5), vermal Crus I (3), vermal Crus II (2), left DN (2), left FN (2) | | | | | | |
|  | VI | right | 25.0 | -61.0 | -16.0 | 10997 | <0.001 | 6919 |
|  | VI | right | 31.0 | -55.0 | -19.0 |  | <0.001 | 6890 |
|  | V | right | 19.0 | -52.0 | -13.0 |  | <0.001 | 6636 |
| 2 | Extended cluster | right VTA (70), left VTA (67) | | | | | | |
|  | VTA | left | -2.0 | -16.0 | -14.5 | 137 | 0.001 | 3389 |
|  | VTA | right | 8.5 | -19.0 | -13.0 |  | 0.001 | 3202 |
| *Figure 4C: US post CS+ > no US post CS- during acquisition, t-test, TFCE, p < 0.05, FWE corr.* | | | | | | | | |
| 1 | Extended cluster | left Crus I (4514), white matter (4220), left VI (3448), right VI (3362), right Crus I (2954), left Crus II (2681), right Crus II (2223), right V (1615), left VIIb (1503), right VIIb (1494), right VIIIa (1382), right I-IV (1368), left V (1251), left I-IV (1147), left VIIIa (1102), right VIIIb (1054), left VIIIb (889), right IX (816), vermal VI (806), left IX (723), vermal VIIIa (452), left DN (308), vermal IX (297), right DN (283), left X (267), right X (252), vermal VIIIb (226), vermal Crus II (168), vermal X (98), vermal VIIb (69), left IN (24), right IN (24), left FN (9), vermal Crus I (8), right FN (8) | | | | | | |
|  | VI | Right | 31.0 | -70.0 | -22.0 | 41045 | <0.001 | 16742 |
|  | VIIb | Right | 20.5 | -70.0 | -49.0 |  | <0.001 | 16480 |
|  | VI | Right | 23.5 | -68.5 | -28.0 |  | <0.001 | 16166 |
| 2 | VTA | Left | -0.5 | -16.0 | -14.5 | 156 | 0.001 | 13357 |
|  | VTA | Right | 7.0 | -16.0 | -10.0 |  | 0.001 | 13090 |
| *Figure 5A: CS+ > CS- during extinction, t-test, TFCE, p < 0.05, FWE corr.* | | | | | | | | |
|  | No significant voxels | | | | | | | |
| *Figure 5B: CS+ x prediction during extinction, t-test, TFCE, p < 0.05, FWE corr.* | | | | | | | | |
| 1 | Extended cluster | left VTA (72), right VTA (63) | | | | | | |
|  | VTA | right | 7.0 | -16.0 | -11.5 | 135 | 0.002 | 2603 |
|  | VTA | left | -3.5 | -16.0 | -13.0 |  | 0.003 | 2495 |
| 2 | Extended cluster | right VI (894), left VI (805), right V (410), left Crus I (197), vermal VI (167), left V (40), right I-IV (17), white matter (14), right Crus I (5), left I-IV (3) | | | | | | |
|  | VI | left | -27.5 | -67.0 | -19.0 | 2552 | 0.004 | 2328 |
|  | VI | right | 31.0 | -65.5 | -19.0 |  | 0.004 | 2298 |
|  | VI | right | 31.0 | -55.0 | -19.0 |  | 0.004 | 2295 |
| 3 | I-IV | left | -3.5 | -46.0 | -4.0 | 10 | 0.009 | 1990 |
| 4 | V | left | -17.0 | -44.5 | -13.0 | 1 | 0.029 | 1457 |
| 5 | I-IV | right | 4.0 | -53.5 | -10.0 | 4 | 0.05 | 1223 |
| *Figure 5C: No US post CS+ > no US post CS- during extinction, t-test, TFCE, p < 0.05, FWE corr.* | | | | | | | | |
|  | No significant voxels | | | | | | | |
