# Supplementary material for: Human cerebellum and ventral tegmental area interact during extinction of learned fear
Source: eLife. 2026 Jul 13;14:RP105399. doi: 10.7554/eLife.105399 (PMC13363218; doi:10.7554/eLife.105399)
Supplement: Supplementary file 9. — Clusters were identified in the cerebellar cortex, deep cerebellar nuclei (DCN), and ventral tegmental area (VTA). Up to three local maxima per cluster are reported, separated by at least 8 mm. Coordinates are given in MNI space (x, y, z). Cluster size is reported as number of voxels (voxel volume = 3.375 mm³). US: unconditioned stimulus; CS: conditioned stimulus; VTA: ventral tegmental area; DCN: deep cerebellar nuclei; DN: dentate nucleus; IN: interposed nucleus; FN: fastigial nucleus; MNI: Montreal Neurological Institute standard brain; t: t-statistic; punc: uncorrected p-value. [file elife-105399-supp9.docx]

## Supplementary fMRI results

### fMRI activation cluster tables

#### *fMRI activations related to the prediction and presentation of the US. Uncorrected.*

***Supplementary file 9:*** *fMRI activation clusters (p < 0.05, uncorrected) related to prediction, presentation and omission of the unconditioned stimulus (US) during acquisition and extinction training (Figure 4 and 5). Clusters were identified in the cerebellar cortex, deep cerebellar nuclei (DCN), and ventral tegmental area (VTA). Up to three local maxima per cluster are reported, separated by at least 8 mm. Coordinates are given in MNI space (x, y, z). Cluster size is reported as number of voxels (voxel volume = 3.375 mm³). US: unconditioned stimulus; CS: conditioned stimulus; VTA: ventral tegmental area; DCN: deep cerebellar nuclei; DN: dentate nucleus; IN: interposed nucleus; FN: fastigial nucleus; MNI: Montreal Neurological Institute standard brain; t: t-statistic; punc: uncorrected p-value.*

| **Index** | **Location (lobule, DCN, VTA)** | **Side** | **MNI coordinates/mm** | | | | **Cluster size (number of voxels)** | **p_unc_** | **t** |
| --- | --- | --- | --- | --- | --- | --- | --- | --- | --- |
|  |  |  | **x** | | **y** | **z** |  |  |  |
| *Figure 4A: CS+ > CS- during acquisition, t-test, p < 0.05, uncorrected* | | | | | | | | | |
| 1 | Extended cluster | right VI (701), right Crus I (259), white matter (4), right V (2), right VIIIa (2),  right X (1) | | | | | | | |
|  | VI | right | 35.5 | | -53.5 | -29.5 | 969 | <0.001 | 5.05 |
|  | VI | right | 34.0 | | -34.0 | -35.5 |  | <0.001 | 4.13 |
|  | VI | right | 28.0 | | -65.5 | -22.0 |  | 0.001 | 3.21 |
| 2 | Extended cluster | left VI (425), left Crus I (386), left Crus II (2) | | | | | | | |
|  | VI | left | -29.0 | | -62.5 | -22.0 | 813 | <0.001 | 4.48 |
|  | Crus I | left | -47.0 | | -44.5 | -32.5 |  | <0.001 | 3.82 |
|  | VI | left | -35.0 | | -55.0 | -28.0 |  | <0.001 | 3.70 |
| 3 | Extended cluster | right VTA (62), left VTA (47) | | | | | | | |
|  | VTA | right | 4.0 | | -16.0 | -13.0 | 109 | <0.001 | 4.31 |
|  | VTA | left | -6.5 | | -17.5 | -11.5 |  | <0.001 | 4.30 |
| 4 | Extended cluster | left Crus I (346), left VI (144), left Crus II (131), vermal VI (96), right VI (51),  white matter (6) | | | | | | | |
|  | VI | right | 8.5 | | -76.0 | -16.0 | 774 | <0.001 | 3.97 |
|  | Crus I | left | -18.5 | | -80.5 | -31.0 |  | <0.001 | 3.56 |
|  | Crus II | left | -5.0 | | -77.5 | -38.5 |  | 0.007 | 2.57 |
| 5 | Extended cluster | left VIIb (369), left Crus II (44), left VIIIa (30) | | | | | | | |
|  | VIIb | left | -33.5 | | -64.0 | -58.0 | 443 | 0.001 | 3.40 |
|  | VIIb | left | -42.5 | | -59.5 | -55.0 |  | 0.001 | 3.23 |
|  | VIIb | left | -38.0 | | -52.0 | -50.5 |  | 0.004 | 2.78 |
| 6 | Extended cluster | right VIIb (150), right VIIIa (146) | | | | | | | |
|  | VIIIa | right | 34.0 | | -58.0 | -55.0 | 296 | 0.001 | 3.39 |
|  | VIIb | right | 40.0 | | -50.5 | -53.5 |  | 0.001 | 3.17 |
| 7 | Extended cluster | left IX (117), vermal IX (63), white matter (14), left VIIIb (4), vermal X (4) | | | | | | | |
|  | IX | left | -8.0 | | -56.5 | -40.0 | 202 | 0.001 | 3.18 |
|  | IX | vermal | -2.0 | | -55.0 | -32.5 |  | 0.008 | 2.51 |
| 8 | Extended cluster | right VIIIa (94), right VIIb (46) | | | | | | | |
|  | VIIIa | right | 8.5 | | -71.5 | -53.5 | 140 | 0.002 | 2.99 |
|  | VIIIa | right | 22.0 | | -65.5 | -56.5 |  | 0.003 | 2.87 |
| 9 | Extended cluster | left VIIIa (44), left VIIb (15) | | | | | | | |
|  | VIIb | left | -18.5 | | -71.5 | -58.0 | 59 | 0.003 | 2.94 |
|  | VIIIa | left | -11.0 | | -68.5 | -56.5 |  | 0.033 | 1.87 |
| 10 | I-IV | left | -0.5 | | -49.0 | -20.5 | 60 | 0.005 | 2.69 |
| 11 | IX | right | 10.0 | | -58.0 | -47.5 | 75 | 0.005 | 2.68 |
| 12 | V | left | -0.5 | | -62.5 | -1.0 | 19 | 0.008 | 2.52 |
| 13 | Crus II | right | 4.0 | | -83.5 | -40.0 | 24 | 0.011 | 2.38 |
| 14 | white matter | | 28.0 | | -52.0 | -46.0 | 16 | 0.014 | 2.28 |
| 15 | Crus II | right | 46.0 | | -59.5 | -50.5 | 14 | 0.015 | 2.23 |
| 16 | VIIIb | right | 20.5 | | -59.5 | -49.0 | 33 | 0.018 | 2.16 |
| 17 | VIIIb | left | -12.5 | | -58.0 | -49.0 | 10 | 0.028 | 1.97 |
| 18 | X | left | -20.0 | | -34.0 | -47.5 | 3 | 0.032 | 1.90 |
| 19 | Crus II | right | 10.0 | | -89.5 | -37.0 | 2 | 0.033 | 1.87 |
| 20 | VIIIa | left | -9.5 | | -70.0 | -55.0 | 2 | 0.033 | 1.87 |
| 21 | IX | right | 8.5 | | -49.0 | -55.0 | 2 | 0.04 | 1.78 |
| 22 | VIIIb | left | -20.0 | | -37.0 | -50.5 | 1 | 0.042 | 1.76 |
| 23 | IX | right | 10.0 | | -55.0 | -55.0 | 4 | 0.042 | 1.76 |
| 24 | white matter | | 10.0 | | -71.5 | -35.5 | 4 | 0.043 | 1.75 |
| 25 | VIIIa | right | 31.0 | | -46.0 | -55.0 | 1 | 0.044 | 1.73 |
| 26 | white matter | | -9.5 | | -50.5 | -28.0 | 1 | 0.045 | 1.73 |
| 27 | I-IV | left | -2.0 | | -56.5 | 0.5 | 1 | 0.045 | 1.73 |
| 28 | Crus I | right | 26.5 | | -83.5 | -22.0 | 1 | 0.046 | 1.71 |
| 29 | Crus II | left | -41.0 | | -46.0 | -46.0 | 2 | 0.046 | 1.71 |
| 30 | VIIIa | left | -6.5 | | -71.5 | -52.0 | 1 | 0.047 | 1.71 |
| 31 | V | left | -29.0 | | -31.0 | -31.0 | 1 | 0.047 | 1.70 |
| *Figure 4B: CS+ x prediction during acquisition, t-test, p < 0.05, uncorrected* | | | | | | | | | |
| 1 | Extended cluster | left VI (2607), right VI (2099), left Crus I (1857), right V (1449), white matter (1256), left V (944), right I-IV (886), right Crus I (780), left Crus II (651), left I-IV (510), vermal VI (436), left VIIb (182), right Crus II (181), vermal IX (140), left VIIIa (120), vermal VIIIa (111), right VIIb (110), left IX (82), right IX (80), right DN (77), vermal VIIIb (61), vermal X (53), right VIIIa (39), vermal VIIb (38), right VIIIb (27), left DN (19), right X (13), vermal Crus II (12), right FN (7), vermal Crus I (4), left FN (4), left IN (3) | | | | | | | |
|  | VI | right | | 25.0 | -59.5 | -16.0 | 14838 | <0.001 | 6.47 |
|  | VI | right | | 31.0 | -55.0 | -19.0 |  | <0.001 | 6.32 |
|  | V | right | | 1.0 | -62.5 | -1.0 |  | <0.001 | 6.15 |
| 2 | Extended cluster | right VTA (72), left VTA (69) | | | | | | | |
|  | VTA | left | -2.0 | | -20.5 | -17.5 | 141 | <0.001 | 5.05 |
|  | VTA | left | -2.0 | | -16.0 | -8.5 |  | <0.001 | 4.78 |
|  | VTA | right | 8.5 | | -19.0 | -13.0 |  | 0.001 | 3.45 |
| 3 | Extended cluster | right Crus II (258), right VIIb (210), right Crus I (14), right VIIIa (11) | | | | | | | |
|  | Crus II | right | 43.0 | | -55.0 | -47.5 | 493 | <0.001 | 3.76 |
|  | Crus II | right | 43.0 | | -67.0 | -53.5 |  | 0.003 | 2.85 |
|  | VIIb | right | 31.0 | | -71.5 | -50.5 |  | 0.011 | 2.38 |
| 4 | Extended cluster | left VIIIa (82), left VIIb (12) | | | | | | | |
|  | VIIIa | left | -27.5 | | -56.5 | -56.5 | 94 | 0.002 | 2.99 |
|  | VIIIa | left | -36.5 | | -53.5 | -58.0 |  | 0.04 | 1.78 |
| 5 | VIIb | left | -30.5 | | -74.5 | -55.0 | 80 | 0.003 | 2.85 |
| 6 | IX | left | -11.0 | | -49.0 | -46.0 | 89 | 0.004 | 2.75 |
| 7 | VIIIb | right | 23.5 | | -46.0 | -58.0 | 16 | 0.007 | 2.56 |
| 8 | Extended cluster | left Crus I (73), left Crus II (9) | | | | | | | |
|  | Crus I | left | -45.5 | | -67.0 | -43.0 | 82 | 0.008 | 2.52 |
|  | Crus I | left | -36.5 | | -61.0 | -40.0 |  | 0.021 | 2.09 |
| 9 | IX | right | 8.5 | | -50.5 | -59.5 | 29 | 0.009 | 2.44 |
| 10 | Crus II | right | 28.0 | | -85.0 | -47.5 | 27 | 0.01 | 2.41 |
| 11 | VIIIa | right | 20.5 | | -62.5 | -52.0 | 45 | 0.012 | 2.35 |
| 12 | VIIIb | left | -24.5 | | -44.5 | -58.0 | 13 | 0.014 | 2.28 |
| 13 | VIIIb | left | -24.5 | | -38.5 | -53.5 | 8 | 0.014 | 2.28 |
| 14 | I-IV | left | -11.0 | | -46.0 | -7.0 | 2 | 0.016 | 2.21 |
| 15 | Crus I | right | 37.0 | | -65.5 | -41.5 | 29 | 0.02 | 2.12 |
| 16 | I-IV | left | -0.5 | | -52.0 | 2.0 | 1 | 0.022 | 2.07 |
| 17 | I-IV | left | -0.5 | | -46.0 | -5.5 | 8 | 0.028 | 1.96 |
| 18 | X | right | 23.5 | | -34.0 | -47.5 | 1 | 0.036 | 1.84 |
| 19 | I-IV | right | 8.5 | | -46.0 | -4.0 | 1 | 0.037 | 1.83 |
| 20 | IX | left | -8.0 | | -52.0 | -59.5 | 3 | 0.038 | 1.81 |
| 21 | VIIb | left | -41.0 | | -52.0 | -55.0 | 2 | 0.039 | 1.80 |
| 22 | VIIIa | right | 10.0 | | -70.0 | -56.5 | 1 | 0.046 | 1.71 |
| 23 | Crus I | left | -53.0 | | -55.0 | -35.5 | 1 | 0.048 | 1.69 |
| 24 | VIIIb | right | 16.0 | | -44.5 | -50.5 | 1 | 0.049 | 1.68 |
| *Figure 4C: US post CS+ > no US post CS- during acquisition, t-test, p < 0.05, uncorrected* | | | | | | | | | |
| 1 | Extended cluster | left Crus I (4378), white matter (3746), left VI (3367), right VI (3353), right Crus I (2720), left Crus II (2536), right Crus II (2069), right V (1592), left VIIb (1456), right VIIb (1455), right VIIIa (1352), right I-IV (1314), left V (1157), left I-IV (1098), right VIIIb (1034), left VIIIa (983), left VIIIb (828), vermal VI (799), right IX (748), left IX (668), vermal VIIIa (452), left DN (308), vermal IX (296), right DN (278), left X (257), right X (246), vermal VIIIb (224), vermal Crus II (168), vermal X (83), vermal VIIb (69), left IN (24), right IN (24), left FN (9), vermal Crus I (8), right FN (8) | | | | | | | |
|  | VIIb | right | 20.5 | | -70.0 | -49.0 | 39107 | <0.001 | 10.43 |
|  | VI | right | 31.0 | | -70.0 | -22.0 |  | <0.001 | 9.82 |
|  | VIIb | left | -17.0 | | -70.0 | -49.0 |  | <0.001 | 8.89 |
| 2 | Extended cluster | right VTA (81), left VTA (75) | | | | | | | |
| 3 | VTA | left | -0.5 | | -16.0 | -14.5 | 156 | <0.001 | 6.77 |
|  | VTA | right | 7.0 | | -16.0 | -10.0 |  | <0.001 | 5.07 |
|  | IX | right | 4.0 | | -46.0 | -43.0 | 5 | 0.012 | 2.35 |
| *Figure 5A: CS+ > CS- during extinction, t-test, p < 0.05, uncorrected* | | | | | | | | | |
| 1 | Extended cluster | right VIIb (96), right VIIIa (53), right Crus II (3) | | | | | | | |
|  | VIIb | right | 34.0 | | -73.0 | -56.5 | 152 | <0.001 | 3.62 |
|  | VIIIa | right | 31.0 | | -58.0 | -52.0 |  | 0.016 | 2.21 |
| 2 | VIIb | right | 23.5 | | -73.0 | -58.0 | 16 | 0.004 | 2.80 |
| 3 | Crus II | left | -15.5 | | -80.5 | -46.0 | 50 | 0.004 | 2.76 |
| 4 | VIIIa | left | -29.0 | | -49.0 | -49.0 | 49 | 0.006 | 2.64 |
| 5 | VIIb | left | -33.5 | | -62.5 | -53.5 | 139 | 0.006 | 2.60 |
| 6 | Crus II | right | 13.0 | | -77.5 | -46.0 | 38 | 0.007 | 2.56 |
| 7 | Extended cluster | white matter (25), right Crus II (20), right Crus I (16), right VI (15), right VIIb (14), right VIIIa (1) | | | | | | | |
|  | Crus II | right | 38.5 | | -49.0 | -44.5 | 91 | 0.008 | 2.53 |
|  | VI | right | 32.5 | | -46.0 | -37.0 |  | 0.012 | 2.36 |
| 8 | Crus I | right | 50.5 | | -61.0 | -41.5 | 12 | 0.015 | 2.25 |
| 9 | VI | left | -33.5 | | -34.0 | -32.5 | 19 | 0.016 | 2.21 |
| 10 | VIIIa | right | 23.5 | | -62.5 | -56.5 | 14 | 0.016 | 2.20 |
| 11 | white matter |  | -18.5 | | -31.0 | -37.0 | 3 | 0.02 | 2.11 |
| 12 | X | right | 16.0 | | -40.0 | -47.5 | 5 | 0.021 | 2.10 |
| 13 | white matter |  | 20.5 | | -34.0 | -40.0 | 12 | 0.024 | 2.04 |
| 14 | VIIb | right | 41.5 | | -50.5 | -53.5 | 2 | 0.026 | 1.99 |
| 15 | white matter |  | -12.5 | | -37.0 | -40.0 | 4 | 0.027 | 1.98 |
| 16 | IX | right | 8.5 | | -53.5 | -55.0 | 13 | 0.027 | 1.98 |
| 17 | VTA | right | 5.5 | | -19.0 | -16.0 | 5 | 0.027 | 1.98 |
| 18 | VIIb | left | -11.0 | | -74.5 | -55.0 | 2 | 0.028 | 1.96 |
| 19 | VI | right | 8.5 | | -76.0 | -16.0 | 6 | 0.029 | 1.94 |
| 20 | VI | left | -15.5 | | -77.5 | -20.5 | 7 | 0.03 | 1.93 |
| 21 | VI | left | -29.0 | | -58.0 | -29.5 | 5 | 0.03 | 1.92 |
| 22 | IX | left | -5.0 | | -52.0 | -44.5 | 4 | 0.034 | 1.87 |
| 23 | IX | left | -6.5 | | -59.5 | -53.5 | 2 | 0.036 | 1.83 |
| 24 | white matter |  | 14.5 | | -40.0 | -40.0 | 1 | 0.036 | 1.83 |
| 25 | VI | right | 37.0 | | -37.0 | -34.0 | 2 | 0.037 | 1.82 |
| 26 | X | left | -27.5 | | -35.5 | -43.0 | 1 | 0.038 | 1.81 |
| 27 | VTA | right | 1.0 | | -19.0 | -10.0 | 3 | 0.038 | 1.81 |
| 28 | VI | left | -20.0 | | -67.0 | -25.0 | 6 | 0.038 | 1.81 |
| 29 | VI | left | -36.5 | | -40.0 | -28.0 | 2 | 0.039 | 1.80 |
| 30 | VIIIb | left | -11.0 | | -56.5 | -56.5 | 5 | 0.039 | 1.80 |
| 31 | VIIIb | vermal | 1.0 | | -65.5 | -49.0 | 2 | 0.042 | 1.76 |
| 32 | VIIb | left | -11.0 | | -77.5 | -52.0 | 1 | 0.042 | 1.76 |
| 33 | Crus I | left | -39.5 | | -50.5 | -40.0 | 1 | 0.043 | 1.75 |
| 34 | VI | right | 16.0 | | -74.5 | -16.0 | 1 | 0.044 | 1.73 |
| 35 | white matter |  | -11.0 | | -41.5 | -38.5 | 1 | 0.047 | 1.70 |
| 36 | Crus II | left | -23.0 | | -79.0 | -50.5 | 1 | 0.047 | 1.70 |
| *Figure 5B: CS+ x prediction during extinction, t-test, p < 0.05, uncorrected* | | | | | | | | | |
| 1 | Extended cluster | left VTA (75), right VTA (74) | | | | | | | |
|  | VTA | right | 7.0 | | -16.0 | -11.5 | 149 | <0.001 | 5.64 |
|  | VTA | left | -5.0 | | -16.0 | -13.0 |  | <0.001 | 4.90 |
| 2 | Extended cluster | left VI (1484), right VI (1377), white matter (711), right V (627), left Crus I (490), right IX (488), left IX (467), right VIIIa (410), right VIIb (380), right VIIIb (246), left VIIIb (234), vermal VI (225), right I-IV (183), right Crus II (178), left I-IV (155), vermal IX (143), vermal X (104), left V (85), right Crus I (84), left X (63), vermal VIIIb (47), right X (46), right DN (37), left DN (32), vermal VIIIa (27), left Crus II (22), left IN (18), left VIIIa (12), left VIIb (7), left FN (6), right FN (3), right IN (2) | | | | | | | |
|  | white matter |  | 5.5 | | -68.5 | -11.5 | 8393 | <0.001 | 5.03 |
|  | VIIIb | left | -18.5 | | -43.0 | -46.0 |  | <0.001 | 4.68 |
|  | VI | left | -5.0 | | -77.5 | -17.5 |  | <0.001 | 4.61 |
| 3 | Extended cluster | left Crus II (198), left VIIb (55), vermal Crus II (9) | | | | | | | |
|  | Crus II | left | -0.5 | | -80.5 | -38.5 | 262 | 0.002 | 3.07 |
|  | VIIb | left | -12.5 | | -76.0 | -47.5 |  | 0.004 | 2.80 |
|  | Crus II | left | -21.5 | | -76.0 | -47.5 |  | 0.024 | 2.03 |
| 4 | I-IV | left | -3.5 | | -46.0 | -4.0 | 25 | 0.002 | 3.04 |
| 5 | Extended cluster | left VIIb (162), left VIIIa (51) | | | | | | | |
|  | VIIb | left | -32.0 | | -61.0 | -55.0 | 213 | 0.006 | 2.65 |
|  | VIIb | left | -38.0 | | -55.0 | -52.0 |  | 0.007 | 2.58 |
|  | VIIb | left | -21.5 | | -71.5 | -56.5 |  | 0.01 | 2.43 |
| 6 | X | right | 20.5 | | -34.0 | -46.0 | 12 | 0.007 | 2.57 |
| 7 | V | left | -17.0 | | -44.5 | -13.0 | 5 | 0.01 | 2.40 |
| 8 | Crus I | right | 41.5 | | -53.5 | -37.0 | 13 | 0.011 | 2.38 |
| 9 | Crus I | right | 40.0 | | -74.5 | -22.0 | 13 | 0.024 | 2.03 |
| 10 | X | left | -18.5 | | -34.0 | -47.5 | 5 | 0.025 | 2.01 |
| 11 | Crus II | left | -32.0 | | -74.5 | -50.5 | 9 | 0.025 | 2.01 |
| 12 | V | left | -21.5 | | -46.0 | -16.0 | 1 | 0.034 | 1.87 |
| 13 | DN | right | 16.0 | | -59.5 | -38.5 | 3 | 0.034 | 1.87 |
| 14 | V | left | -23.0 | | -50.5 | -16.0 | 1 | 0.038 | 1.81 |
| 15 | Crus I | right | 47.5 | | -53.5 | -34.0 | 1 | 0.038 | 1.81 |
| 16 | VI | vermal | 5.5 | | -71.5 | -25.0 | 4 | 0.038 | 1.81 |
| 17 | Crus II | left | -33.5 | | -77.5 | -53.5 | 2 | 0.039 | 1.80 |
| 18 | Crus II | right | 34.0 | | -76.0 | -52.0 | 3 | 0.039 | 1.79 |
| 19 | white matter |  | -17.0 | | -31.0 | -35.5 | 2 | 0.039 | 1.79 |
| 20 | I-IV | right | 2.5 | | -41.5 | -16.0 | 1 | 0.041 | 1.77 |
| 21 | Crus I | left | -45.5 | | -43.0 | -31.0 | 1 | 0.044 | 1.74 |
| *Figure 5C: No US post CS+ > no US post CS- during extinction, t-test, p <0.05, uncorrected* | | | | | | | | | |
| 1 | Extended cluster | left Crus I (1015), white matter (539), left VI (451), left Crus II (386), left VIIb (58), left VIIIa (34), left DN (34), left V (22), left IX (8) | | | | | | | |
|  | VI | left | -24.5 | | -55.0 | -31.0 | 2547 | <0.001 | 4.20 |
|  | Crus I | left | -41.0 | | -58.0 | -37.0 |  | <0.001 | 3.84 |
|  | white matter |  | -30.5 | | -52.0 | -37.0 |  | <0.001 | 3.71 |
| 2 | Extended cluster | right V (191), left V (160), left VI (109), vermal VI (97), right VI (86), right Crus I (40), right I-IV (14), white matter (2) | | | | | | | |
|  | V | right | 2.5 | | -67.0 | -5.5 | 699 | <0.001 | 3.73 |
|  | VI | right | 8.5 | | -80.5 | -20.5 |  | 0.001 | 3.33 |
|  | V | left | -6.5 | | -62.5 | -10.0 |  | 0.001 | 3.30 |
| 3 | Extended cluster | right V (207), right VI (199), white matter (194), right I-IV (126), right Crus I (12) | | | | | | | |
|  | V | right | 28.0 | | -41.5 | -28.0 | 738 | 0.001 | 3.33 |
|  | white matter |  | 20.5 | | -40.0 | -31.0 |  | 0.001 | 3.18 |
|  | I-IV | right | 10.0 | | -47.5 | -25.0 |  | 0.002 | 3.14 |
| 4 | Extended cluster | white matter (120), right VIIb (115), right Crus II (68), right VIIIa (58), right Crus I (25) | | | | | | | |
|  | white matter |  | 28.0 | | -49.0 | -41.5 | 386 | 0.001 | 3.17 |
|  | VIIb | right | 38.5 | | -47.5 | -46.0 |  | 0.002 | 3.09 |
|  | VIIIa | right | 32.5 | | -52.0 | -53.5 |  | 0.006 | 2.65 |
| 5 | VIIIb | right | 23.5 | | -50.5 | -56.5 | 38 | 0.002 | 3.07 |
| 6 | Extended cluster | right Crus I (311), right VI (222) | | | | | | | |
|  | VI | right | 22.0 | | -68.5 | -28.0 | 533 | 0.002 | 2.99 |
|  | VI | right | 32.5 | | -67.0 | -26.5 |  | 0.003 | 2.84 |
|  | VI | right | 28.0 | | -59.5 | -28.0 |  | 0.012 | 2.33 |
| 7 | IX | right | 5.5 | | -49.0 | -59.5 | 11 | 0.003 | 2.88 |
| 8 | Extended cluster | white matter (119), left DN (20), left I-IV (15), left IN (13), left V (12) | | | | | | | |
|  | DN | left | -9.5 | | -56.5 | -29.5 | 179 | 0.003 | 2.87 |
|  | I-IV | left | -8.0 | | -47.5 | -25.0 |  | 0.016 | 2.21 |
| 9 | I-IV | right | 14.5 | | -43.0 | -11.5 | 15 | 0.005 | 2.71 |
| 10 | Crus I | left | -51.5 | | -67.0 | -37.0 | 15 | 0.005 | 2.68 |
| 11 | VTA | right | 2.5 | | -16.0 | -13.0 | 19 | 0.008 | 2.53 |
| 12 | VIIIa | right | 17.5 | | -65.5 | -58.0 | 27 | 0.008 | 2.50 |
| 13 | Extended cluster | left VIIb (40), left VIIIa (36) | | | | | | | |
|  | VIIb | left | -38.0 | | -62.5 | -59.5 | 76 | 0.009 | 2.48 |
|  | VIIIa | left | -32.0 | | -52.0 | -56.5 |  | 0.017 | 2.18 |
| 14 | VIIIb | left | -23.0 | | -38.5 | -53.5 | 12 | 0.01 | 2.42 |
| 15 | I-IV | left | -0.5 | | -52.0 | 2.0 | 1 | 0.012 | 2.33 |
| 16 | VIIIa | left | -20.0 | | -64.0 | -59.5 | 35 | 0.013 | 2.29 |
| 17 | VTA | left | -8.0 | | -17.5 | -10.0 | 7 | 0.014 | 2.28 |
| 18 | Crus I | right | 23.5 | | -83.5 | -22.0 | 10 | 0.015 | 2.24 |
| 19 | Crus II | left | -9.5 | | -74.5 | -34.0 | 15 | 0.016 | 2.23 |
| 20 | VIIb | left | -18.5 | | -79.0 | -53.5 | 11 | 0.016 | 2.22 |
| 21 | VIIIa | right | 29.5 | | -62.5 | -53.5 | 8 | 0.017 | 2.19 |
| 22 | VI | right | 28.0 | | -59.5 | -19.0 | 20 | 0.02 | 2.12 |
| 23 | Extended cluster | right IX (18), vermal VIIIb (4), vermal VIIIa (2), right VIIIa (1) | | | | | | | |
|  | IX | right | 5.5 | | -58.0 | -43.0 | 25 | 0.02 | 2.12 |
|  | VIIIa | right | 7.0 | | -67.0 | -46.0 |  | 0.042 | 1.76 |
| 24 | V | left | -21.5 | | -49.0 | -16.0 | 3 | 0.021 | 2.10 |
| 25 | Crus II | right | 4.0 | | -77.5 | -37.0 | 8 | 0.022 | 2.07 |
| 26 | white matter |  | 5.5 | | -71.5 | -11.5 | 1 | 0.024 | 2.03 |
| 27 | VIIb | right | 20.5 | | -77.5 | -55.0 | 5 | 0.025 | 2.01 |
| 28 | VI | right | 20.5 | | -74.5 | -17.5 | 1 | 0.027 | 1.97 |
| 29 | VI | vermal | 2.5 | | -62.5 | -25.0 | 16 | 0.029 | 1.94 |
| 30 | V | left | -21.5 | | -41.5 | -23.5 | 6 | 0.03 | 1.93 |
| 31 | VI | right | 23.5 | | -71.5 | -17.5 | 3 | 0.032 | 1.90 |
| 32 | VIIIb | right | 14.5 | | -56.5 | -52.0 | 4 | 0.034 | 1.86 |
| 33 | VIIb | left | -5.0 | | -71.5 | -43.0 | 3 | 0.036 | 1.83 |
| 34 | V | left | -20.0 | | -53.5 | -16.0 | 6 | 0.037 | 1.82 |
| 35 | VI | right | 20.5 | | -59.5 | -28.0 | 5 | 0.038 | 1.81 |
| 36 | VIIb | right | 13.0 | | -71.5 | -53.5 | 9 | 0.039 | 1.80 |
| 37 | V | left | -23.0 | | -34.0 | -22.0 | 2 | 0.039 | 1.80 |
| 38 | I-IV | right | 10.0 | | -50.5 | -5.5 | 1 | 0.041 | 1.78 |
| 39 | VIIb | left | -42.5 | | -52.0 | -53.5 | 1 | 0.041 | 1.77 |
| 40 | DN | right | 14.5 | | -62.5 | -37.0 | 1 | 0.044 | 1.73 |
| 41 | white matter |  | -11.0 | | -38.5 | -35.5 | 1 | 0.046 | 1.72 |
| 42 | VIIIb | left | -21.5 | | -52.0 | -58.0 | 1 | 0.047 | 1.70 |
| 43 | I-IV | right | 5.5 | | -53.5 | 0.5 | 1 | 0.048 | 1.69 |
