# Supplementary material for: Human cerebellum and ventral tegmental area interact during extinction of learned fear
Source: eLife. 2026 Jul 13;14:RP105399. doi: 10.7554/eLife.105399 (PMC13363218; doi:10.7554/eLife.105399)
Supplement: Supplementary file 10. — Clusters were identified in the cerebellar cortex, deep cerebellar nuclei (DCN), and ventral tegmental area (VTA) using threshold-free cluster enhancement (TFCE) with family-wise error (FWE) correction (p<0.05). Up to three local maxima per cluster are reported, separated by at least 8 mm. Coordinates are given in MNI space (x, y, z). Cluster size is reported as number of voxels (voxel volume = 3.375 mm³). US: unconditioned stimulus; CS: conditioned stimulus; VTA: ventral tegmental area; DCN: deep cerebellar nuclei; DN: dentate nucleus; IN: interposed nucleus; FN: fastigial nucleus; MNI: Montreal Neurological Institute standard brain; TFCE t: threshold-free cluster-enhanced t-statistic; pFWE: family-wise error-corrected p-value. [file elife-105399-supp10.docx]

## Supplementary fMRI results

### fMRI activation cluster tables

#### *fMRI activations related to the unexpected omission of the US. TFCE and FWE corrected.*

***Supplementary file 10:*** *fMRI activation clusters related to the unexpected omission of the unconditioned stimulus (US) during extinction training (Figure 6 and 7). Clusters were identified in the cerebellar cortex, deep cerebellar nuclei (DCN), and ventral tegmental area (VTA) using threshold-free cluster enhancement (TFCE) with family-wise error (FWE) correction (p < 0.05). Up to three local maxima per cluster are reported, separated by at least 8 mm. Coordinates are given in MNI space (x, y, z). Cluster size is reported as number of voxels (voxel volume = 3.375 mm³). US: unconditioned stimulus; CS: conditioned stimulus; VTA: ventral tegmental area; DCN: deep cerebellar nuclei; DN: dentate nucleus; IN: interposed nucleus; FN: fastigial nucleus; MNI: Montreal Neurological Institute standard brain; TFCE t: threshold-free cluster-enhanced t-statistic; pFWE: family-wise error-corrected p-value.*

| **Index** | **Location (lobule, DCN, VTA)** | **Side** | **MNI coordinates/mm** | | | **Cluster size (number of voxels)** | **p_FWE_** | **TFCE t** |
| --- | --- | --- | --- | --- | --- | --- | --- | --- |
|  |  |  | **x** | **y** | **z** |  |  |  |
| *Figure 6A: First 3 no US post CS+ > no US post CS- during extinction, t-test, TFCE, p < 0.05, FWE corr.* | | | | | | | | |
| 1 | Extended cluster | left Crus I (2540), left Crus II (2266), white matter (1957), left VI (1531), right VI (1403), right Crus I (1144), left VIIb (811), left DN (273), right V (240), right I-IV (208), left I-IV (185), right DN (154), left VIIIa (138), vermal VI (126), vermal VIIIa (115), left V (114), left IX (93), right Crus II (79), left VIIIb (57), vermal IX (29), right IX (29), left IN (20), left X (15), vermal VIIb (9), vermal Crus I (5), vermal Crus II (4), vermal VIIIb (4), right IN (4), vermal X (3), right VIIb (1), left FN (1) | | | | | | |
|  | Crus I | left | -38.0 | -53.5 | -35.5 | 13558 | <0.001 | 3262 |
|  | VI | left | -20.0 | -68.5 | -26.5 |  | 0.001 | 3214 |
|  | VI | left | -24.5 | -50.5 | -31.0 |  | 0.001 | 3073 |
| 2 | Extended cluster | right VIIb (193), right Crus II (113), right VIIIa (11) | | | | | | |
|  | Crus II | right | 7.0 | -79.0 | -43.0 | 317 | 0.007 | 2053 |
|  | VIIb | right | 22.0 | -71.5 | -52.0 |  | 0.013 | 1773 |
|  | VIIb | right | 14.5 | -76.0 | -50.5 |  | 0.014 | 1746 |
| 3 | I-IV | right | 4.0 | -52.0 | 0.5 | 2 | 0.018 | 1642 |
| 4 | Extended cluster | left VTA (35), right VTA (22) | | | | | | |
|  | VTA | right | 8.5 | -17.5 | -10.0 | 57 | 0.022 | 1562 |
|  | VTA | left | -8.0 | -17.5 | -10.0 |  | 0.022 | 1550 |
|  | VTA | left | -0.5 | -16.0 | -14.5 |  | 0.031 | 1413 |
| 5 | Extended cluster | right VIIb (197), right Crus II (171), right VIIIa (146), white matter (31), right Crus I (24), right X (11), right VI (9) | | | | | | |
|  | VIIIa | right | 35.5 | -52.0 | -52.0 | 589 | 0.022 | 1551 |
|  | Crus II | right | 43.0 | -49.0 | -46.0 |  | 0.027 | 1470 |
|  | VIIb | right | 35.5 | -44.5 | -43.0 |  | 0.028 | 1451 |
| 6 | VI | right | 20.5 | -74.5 | -17.5 | 1 | 0.023 | 1545 |
| 7 | I-IV | right | 5.5 | -49.0 | -1.0 | 3 | 0.023 | 1545 |
| 8 | I-IV | left | -5.0 | -49.0 | -1.0 | 2 | 0.023 | 1545 |
| 9 | VTA | right | 1.0 | -20.5 | -8.5 | 5 | 0.031 | 1412 |
| 10 | VTA | left | -0.5 | -20.5 | -17.5 | 2 | 0.036 | 1348 |
| 11 | VI | left | -8.0 | -67.0 | -10.0 | 1 | 0.039 | 1315 |
| 12 | Crus II | right | 28.0 | -83.5 | -46.0 | 52 | 0.042 | 1281 |
| *Figure 6B: First 3 no US post CS+ > no US post CS- during recall, t-test, TFCE, p < 0.05, FWE corr.* | | | | | | | | |
| 1 | Extended cluster | left Crus I (1340), left VI (1298), left Crus II (599), left VIIb (142), vermal VI (132), white matter (55), left DN (20), left VIIIa (17), right VI (9), left V (6), vermal Crus I (4) | | | | | | |
|  | VI | left | -21.5 | -71.5 | -28.0 | 3622 | 0.001 | 2073 |
|  | VI | left | -29.0 | -67.0 | -26.5 |  | 0.002 | 2046 |
|  | VI | left | -8.0 | -76.0 | -26.5 |  | 0.002 | 1988 |
| 2 | Extended cluster | vermal VIIIa (140), vermal VIIIb (138), vermal IX (137), left IX (55), left VIIIb (43), white matter (31), left VIIIa (9), vermal X (8), left DN (1) | | | | | | |
|  | IX | vermal | -0.5 | -59.5 | -35.5 | 562 | 0.021 | 1318 |
|  | VIIIb | vermal | -0.5 | -65.5 | -44.5 |  | 0.028 | 1245 |
|  | VIIIb | left | -9.5 | -61.0 | -43.0 |  | 0.028 | 1243 |
| 3 | Extended cluster | right VI (344), white matter (159), right DN (46), right Crus I (30), right IN (8) | | | | | | |
|  | VI | right | 34.0 | -55.0 | -29.5 | 587 | 0.021 | 1317 |
|  | VI | right | 32.5 | -55.0 | -20.5 |  | 0.024 | 1291 |
|  | VI | right | 20.5 | -58.0 | -28.0 |  | 0.024 | 1289 |
| 4 | I-IV | right | 14.5 | -43.0 | -11.5 | 43 | 0.024 | 1291 |
| 5 | V | right | 20.5 | -53.5 | -13.0 | 7 | 0.024 | 1288 |
| 6 | Extended cluster | right VIIIa (295), right VIIb (230), right Crus II (94), right VIIIb (66), white matter (16), vermal VIIIa (2) | | | | | | |
|  | VIIb | right | 19.0 | -73.0 | -49.0 | 703 | 0.027 | 1256 |
|  | VIIIa | right | 23.5 | -64.0 | -49.0 |  | 0.027 | 1249 |
|  | VIIIa | right | 32.5 | -52.0 | -52.0 |  | 0.03 | 1224 |
| 7 | VTA | right | 2.5 | -17.5 | -14.5 | 39 | 0.033 | 1192 |
| 8 | Crus I | left | -36.5 | -80.5 | -25.0 | 1 | 0.035 | 1178 |
| 9 | Extended cluster | left VIIb (194), left VIIIa (43), left Crus II (27), left VIIIb (1) | | | | | | |
|  | VIIb | left | -36.5 | -47.5 | -50.5 | 265 | 0.037 | 1164 |
|  | VIIb | left | -38.0 | -55.0 | -56.5 |  | 0.037 | 1162 |
|  | Crus II | left | -42.5 | -62.5 | -50.5 |  | 0.042 | 1130 |
| 10 | VI | right | 23.5 | -77.5 | -19.0 | 2 | 0.039 | 1151 |
| 11 | V | right | 8.5 | -56.5 | -4.0 | 2 | 0.039 | 1151 |
| 12 | Extended cluster | right IX (86), white matter (32), right VIIIb (23), right X (17) | | | | | | |
|  | IX | right | 13.0 | -53.5 | -46.0 | 158 | 0.039 | 1151 |
|  | VIIIb | right | 19.0 | -43.0 | -49.0 |  | 0.045 | 1112 |
| 13 | VIIIb | left | -17.0 | -52.0 | -50.5 | 41 | 0.04 | 1140 |
| 14 | VI | right | 13.0 | -76.0 | -16.0 | 3 | 0.042 | 1127 |
| 15 | I-IV | right | 4.0 | -46.0 | -5.5 | 52 | 0.044 | 1118 |
| 16 | VTA | left | -8.0 | -19.0 | -11.5 | 1 | 0.044 | 1115 |
| 17 | Extended cluster | left X (49), white matter (16), left VIIIb (12) | | | | | | |
|  | VIIIb | left | -24.5 | -38.5 | -47.5 | 77 | 0.045 | 1111 |
|  | X | left | -15.5 | -40.0 | -44.5 |  | 0.045 | 1110 |
| 18 | VI | right | 29.5 | -47.5 | -20.5 | 1 | 0.046 | 1102 |
| 19 | VI | right | 20.5 | -71.5 | -16.0 | 1 | 0.046 | 1102 |
| 20 | V | right | 23.5 | -49.0 | -16.0 | 1 | 0.046 | 1102 |
| 21 | V | left | -17.0 | -46.0 | -13.0 | 1 | 0.048 | 1092 |
| 22 | X | right | 22.0 | -38.5 | -46.0 | 4 | 0.048 | 1090 |
| *Figure 6C: First 3 no US post CS+ > no US post CS- during reacquisition, t-test, TFCE, p < 0.05, FWE corr.* | | | | | | | | |
| 1 | Extended cluster | right VTA (74), left VTA (71) | | | | | | |
|  | VTA | left | -2.0 | -17.5 | -7.0 | 145 | 0.001 | 7243 |
|  | VTA | right | 8.5 | -16.0 | -11.5 |  | 0.001 | 6425 |
|  | VTA | left | -0.5 | -20.5 | -17.5 |  | 0.002 | 4461 |
| 2 | Extended cluster | left Crus I (3033), left Crus II (2504), left VI (2143), right VI (2121), right Crus I (1081), left VIIb (982), white matter (896), right VIIb (878), left IX (695), right VIIIa (604), right Crus II (582), right IX (351), vermal IX (252), left VIIIb (246), right X (244), vermal VI (176), left X (175), right I-IV (153), right DN (152), left DN (151), left I-IV (148), right V (130), right VIIIb (121), left VIIIa (75), left V (61), vermal VIIIb (54), vermal VIIIa (47), vermal X (45), right IN (6), vermal Crus I (5), vermal Crus II (2), left FN (2), left IN (1) | | | | | | |
|  | Crus I | left | -9.5 | -82.0 | -28.0 | 18116 | 0.001 | 6274 |
|  | VI | left | -26.0 | -65.5 | -28.0 |  | 0.001 | 6189 |
|  | VI | left | -29.0 | -59.5 | -32.5 |  | 0.001 | 6171 |
| 3 | white matter | | -9.5 | -38.5 | -31.0 | 20 | 0.005 | 2394 |
| 4 | white matter | | -12.5 | -37.0 | -41.5 | 4 | 0.01 | 988 |
| *Figure 6D: First 3 no US post CS+ > no US post CS- during reextinction, t-test, TFCE, p < 0.05, FWE corr.* | | | | | | | | |
| 1 | Extended cluster | left Crus I (1030), left VI (807), left Crus II (63), white matter (1), vermal VI (1) | | | | | | |
|  | VI | left | -8.0 | -76.0 | -26.5 | 1902 | 0.004 | 2229 |
|  | Crus I | left | -15.5 | -79.0 | -25.0 |  | 0.005 | 2151 |
|  | VI | left | -30.5 | -58.0 | -31.0 |  | 0.007 | 2020 |
| 2 | Extended cluster | left VTA (47), right VTA (34) | | | | | | |
|  | VTA | right | 4.0 | -14.5 | -13.0 | 81 | 0.027 | 1432 |
|  | VTA | left | -5.0 | -16.0 | -13.0 |  | 0.028 | 1425 |
| 3 | VTA | right | 4.0 | -14.5 | -7.0 | 4 | 0.034 | 1340 |
| 4 | Crus I | right | 38.5 | -53.5 | -31.0 | 4 | 0.049 | 1194 |
| *Figure 7A: No US post CS+ x prediction error during extinction, t-test, TFCE, p < 0.05, FWE corr.* | | | | | | | | |
| 1 | Extended cluster | left Crus I (2341), left Crus II (1671), left VI (1220), white matter (529), left VIIb (337), left IX (269), vermal VIIIa (135), vermal IX (135), left DN (133), right I-IV (123), left I-IV (122), left VIIIb (64), left X (56), left VIIIa (44), vermal X (33), left V (30), vermal VIIIb (26), right V (23), vermal VI (15), left IN (10), vermal VIIb (9), vermal Crus I (6), vermal Crus II (4), right FN (4), left FN (2) | | | | | | |
|  | Crus I | left | -11.0 | -79.0 | -26.5 | 7341 | 0.002 | 2645 |
|  | VI | left | -23.0 | -68.5 | -28.0 |  | 0.003 | 2422 |
|  | Crus I | left | -20.0 | -76.0 | -31.0 |  | 0.003 | 2410 |
| 2 | Extended cluster | left VTA (54), right VTA (52) | | | | | | |
|  | VTA | left | -5.0 | -17.5 | -13.0 | 106 | 0.011 | 1808 |
|  | VTA | right | 5.5 | -16.0 | -14.5 |  | 0.013 | 1770 |
| 3 | I-IV | right | 5.5 | -49.0 | -1.0 | 3 | 0.021 | 1569 |
| 4 | Extended cluster | right V (9), left V (2) | | | | | | |
|  | V | right | 1.0 | -62.5 | -1.0 | 11 | 0.025 | 1496 |
|  | V | right | 5.5 | -67.0 | -8.5 |  | 0.025 | 1484 |
| 5 | I-IV | left | -5.0 | -49.0 | -1.0 | 3 | 0.026 | 1482 |
| 6 | Extended cluster | right Crus I (600), right VI (246), right Crus II (16), vermal Crus II (1) | | | | | | |
|  | VI | right | 37.0 | -50.5 | -31.0 | 863 | 0.034 | 1368 |
|  | Crus I | right | 38.5 | -76.0 | -25.0 |  | 0.035 | 1356 |
|  | Crus I | right | 13.0 | -80.5 | -26.5 |  | 0.036 | 1344 |
| 7 | I-IV | right | 4.0 | -52.0 | 0.5 | 1 | 0.039 | 1315 |
| 8 | Crus I | left | -45.5 | -43.0 | -31.0 | 1 | 0.043 | 1275 |
| 9 | DN | right | 16.0 | -58.0 | -34.0 | 136 | 0.044 | 1263 |
| 10 | Crus I | right | 23.5 | -83.5 | -22.0 | 2 | 0.046 | 1251 |
| 11 | VI | right | 16.0 | -80.5 | -19.0 | 4 | 0.046 | 1251 |
| 12 | V | right | 29.5 | -44.5 | -22.0 | 41 | 0.046 | 1248 |
|  | VI | right | 35.5 | -52.0 | -22.0 |  | 0.049 | 1226 |
| *Figure 7B: No US post CS+ x prediction error during recall test, t-test, TFCE, p <0.05, FWE corr.* | | | | | | | | |
| 1 | Extended cluster | left VI (357), left Crus I (325), white matter (2) | | | | | | |
|  | VI | left | -33.5 | -55.0 | -32.5 | 684 | 0.003 | 1981 |
|  | VI | left | -29.0 | -64.0 | -29.5 |  | 0.006 | 1772 |
|  | VI | left | -38.0 | -40.0 | -35.5 |  | 0.043 | 1198 |
| 2 | Extended cluster | left VI (56), vermal VI (11), left Crus I (9) | | | | | | |
|  | VI | vermal | -3.5 | -79.0 | -19.0 | 76 | 0.038 | 1231 |
|  | VI | left | -12.5 | -80.5 | -20.5 |  | 0.041 | 1213 |
|  | VI | left | -20.0 | -77.5 | -23.5 |  | 0.045 | 1180 |
| 3 | Crus I | left | -14.0 | -79.0 | -29.5 | 60 | 0.043 | 1193 |
| 4 | Crus II | left | -14.0 | -79.0 | -35.5 | 2 | 0.05 | 1151 |
| *Figure 7C: No US post CS+ x prediction error during reacquisition, t-test, TFCE, p < 0.05, FWE corr.* | | | | | | | | |
|  | No significant voxels | | | | | | | |
| *Figure 7D: No US post CS+ x prediction error during reextinction, t-test, TFCE, p < 0.05, FWE corr.* | | | | | | | | |
|  | No significant voxels | | | | | | | |
