# Supplementary material for: Human cerebellum and ventral tegmental area interact during extinction of learned fear
Source: eLife. 2026 Jul 13;14:RP105399. doi: 10.7554/eLife.105399 (PMC13363218; doi:10.7554/eLife.105399)
Supplement: Supplementary file 11. — Clusters were identified in the cerebellar cortex, deep cerebellar nuclei (DCN), and ventral tegmental area (VTA). Up to three local maxima per cluster are reported, separated by at least 8 mm. Coordinates are given in MNI space (x, y, z). Cluster size is reported as number of voxels (voxel volume = 3.375 mm³). US: unconditioned stimulus; CS: conditioned stimulus; VTA: ventral tegmental area; DCN: deep cerebellar nuclei; DN: dentate nucleus; IN: interposed nucleus; FN: fastigial nucleus; MNI: Montreal Neurological Institute standard brain; t: t-statistic; punc: uncorrected p-value. [file elife-105399-supp11.docx]

## Supplementary fMRI results

### fMRI activation cluster tables

#### *fMRI activations related to the unexpected omission of the US. Uncorrected.*

***Supplementary file 11:*** *fMRI activation clusters (p < 0.05, uncorrected) related to the unexpected omission of the unconditioned stimulus (US) during extinction training (Figure 6 and 7). Clusters were identified in the cerebellar cortex, deep cerebellar nuclei (DCN), and ventral tegmental area (VTA). Up to three local maxima per cluster are reported, separated by at least 8 mm. Coordinates are given in MNI space (x, y, z). Cluster size is reported as number of voxels (voxel volume = 3.375 mm³). US: unconditioned stimulus; CS: conditioned stimulus; VTA: ventral tegmental area; DCN: deep cerebellar nuclei; DN: dentate nucleus; IN: interposed nucleus; FN: fastigial nucleus; MNI: Montreal Neurological Institute standard brain; t: t-statistic; punc: uncorrected p-value.*

| **Index** | **Location (lobule, DCN, VTA)** | **Side** | **MNI coordinates/mm** | | | **Cluster size (number of voxels)** | **p_unc_** | **t** |
| --- | --- | --- | --- | --- | --- | --- | --- | --- |
|  |  |  | **x** | **y** | **z** |  |  |  |
| *Figure 6A: First 3 no US post CS+ > no US post CS- during extinction, t-test, p < 0.05, uncorrected* | | | | | | | | |
| 1 | Extended cluster | left Crus I (3015), white matter (2887), left Crus II (2640), right VI (1855), left VI (1794), right Crus I (1739), left VIIb (1040), right Crus II (838), right VIIb (580), right V (447), right I-IV (333), right VIIIa (327), left DN (289), left V (283), left I-IV (272), left VIIIa (258), left IX (255), vermal VI (232), vermal VIIIa (227), right DN (225), right VIIIb (166), left VIIIb (134), right IX (103), vermal IX (75), right X (57), vermal VIIIb (50), left X (41), vermal Crus II (29), left IN (22), vermal VIIb (17), right IN (11), vermal X (9), vermal Crus I (7), left FN (1) | | | | | | |
|  | VI | left | -20.0 | -68.5 | -26.5 | 20258 | <0.001 | 6.92 |
|  | Crus I | left | -38.0 | -53.5 | -35.5 |  | <0.001 | 6.77 |
|  | Crus II | left | -29.0 | -77.5 | -41.5 |  | <0.001 | 6.08 |
| 2 | Extended cluster | left VTA (53), right VTA (49) | | | | | | |
|  | VTA | left | -8.0 | -17.5 | -10.0 | 102 | <0.001 | 3.82 |
|  | VTA | right | 8.5 | -17.5 | -10.0 |  | <0.001 | 3.74 |
|  | VTA | left | -0.5 | -16.0 | -14.5 |  | 0.004 | 2.77 |
| 3 | I-IV | left | -5.0 | -49.0 | -1.0 | 6 | 0.012 | 2.34 |
| 4 | I-IV | left | -0.5 | -52.0 | 2.0 | 1 | 0.021 | 2.08 |
| 5 | IX | right | 5.5 | -49.0 | -59.5 | 1 | 0.027 | 1.97 |
| 6 | VIIIb | right | 20.5 | -46.0 | -56.5 | 6 | 0.029 | 1.94 |
| 7 | Crus I | left | -33.5 | -86.5 | -32.5 | 2 | 0.031 | 1.91 |
| 8 | white matter |  | 22.0 | -32.5 | -37.0 | 5 | 0.031 | 1.91 |
| 9 | V | left | -18.5 | -47.5 | -14.5 | 1 | 0.036 | 1.84 |
| 10 | Crus II | left | -26.0 | -89.5 | -37.0 | 3 | 0.038 | 1.81 |
| 11 | white matter |  | 32.5 | -62.5 | -38.5 | 3 | 0.04 | 1.79 |
| *Figure 6B: First 3 no US post CS+ > no US post CS- during recall, t-test, p < 0.05, uncorrected* | | | | | | | | |
| 1 | Extended cluster | left Crus I (1983), left VI (1831), left Crus II (1548), right VI (1164), white matter (1145), left VIIb (683), right VIIIa (557), right VIIb (554), right Crus II (384), left VIIIa (353), right I-IV (350), left VIIIb (343), right VIIIb (335), vermal VIIIa (294), vermal VI (273), right Crus I (205), left IX (197), vermal IX (197), right IX (189), vermal VIIIb (182), left X (162), right V (153), right DN (153), left I-IV (145), left DN (137), right X (121), left V (93), vermal X (25), right IN (21), vermal Crus II (19), vermal VIIb (9), vermal Crus I (7), left FN (3), left IN (1), right FN (1) | | | | | | |
|  | VI | left | -21.5 | -71.5 | -28.0 | 13817 | <0.001 | 5.60 |
|  | VI | left | -8.0 | -76.0 | -26.5 |  | <0.001 | 5.45 |
|  | VI | left | -35.0 | -53.5 | -31.0 |  | <0.001 | 5.37 |
| 2 | Extended cluster | right VTA (51), left VTA (47) | | | | | | |
|  | VTA | right | 2.5 | -17.5 | -14.5 | 98 | <0.001 | 4.75 |
|  | VTA | left | -8.0 | -19.0 | -11.5 |  | <0.001 | 3.57 |
| 3 | I-IV | right | 13.0 | -34.0 | -22.0 | 26 | 0.002 | 3.09 |
| 4 | V | left | -17.0 | -46.0 | -13.0 | 4 | 0.004 | 2.75 |
| 5 | V | left | -14.0 | -50.5 | -10.0 | 6 | 0.01 | 2.44 |
| 6 | Extended cluster | left I-IV (45) | | | | | | |
|  | I-IV | left | -8.0 | -38.5 | -20.5 | 45 | 0.012 | 2.34 |
|  | I-IV | left | -6.5 | -41.5 | -11.5 |  | 0.014 | 2.28 |
| 7 | V | left | -3.5 | -58.0 | -13.0 | 13 | 0.013 | 2.31 |
| 8 | Crus I | right | 47.5 | -53.5 | -44.5 | 5 | 0.017 | 2.18 |
| 9 | IX | right | 5.5 | -58.0 | -55.0 | 8 | 0.022 | 2.07 |
| 10 | VIIIa | right | 10.0 | -68.5 | -53.5 | 6 | 0.032 | 1.90 |
| 11 | VI | right | 23.5 | -71.5 | -17.5 | 1 | 0.036 | 1.84 |
| 12 | IX | left | -6.5 | -56.5 | -53.5 | 5 | 0.037 | 1.83 |
| 13 | VI | right | 10.0 | -74.5 | -14.5 | 1 | 0.038 | 1.81 |
| 14 | V | left | -8.0 | -58.0 | -4.0 | 1 | 0.038 | 1.81 |
| 15 | VI | left | -23.0 | -62.5 | -16.0 | 1 | 0.042 | 1.75 |
| 16 | V | left | -0.5 | -68.5 | -8.5 | 1 | 0.046 | 1.71 |
| 17 | white matter |  | -9.5 | -41.5 | -50.5 | 1 | 0.048 | 1.69 |
| *Figure 6C: First 3 no US post CS+ > no US post CS- reacquisition, t-test, p < 0.05, uncorrected* | | | | | | | | |
| 1 | Extended cluster | left Crus I (2890), left Crus II (2337), left VI (2089), right VI (1996), right Crus I (986), left VIIb (932), right VIIb (789), white matter (747), left IX (650), right VIIIa (546), right Crus II (468), right IX (263), vermal IX (240), right X (240), left VIIIb (225), vermal VI (159), left X (157), left DN (132), right DN (121), left I-IV (106), right I-IV (90), right VIIIb (87), right V (70), left VIIIa (63), vermal VIIIa (41), vermal VIIIb (41), vermal X (37), left V (14), vermal Crus I (5), right IN (4), vermal Crus II (2), left FN (2) | | | | | | |
|  | Crus I | left | -38.0 | -58.0 | -31.0 | 16529 | <0.001 | 9.00 |
|  | Crus I | left | -9.5 | -82.0 | -28.0 |  | <0.001 | 8.92 |
|  | VI | left | -26.0 | -65.5 | -28.0 |  | <0.001 | 8.24 |
| 2 | Extended cluster | right VTA (72), left VTA (67) | | | | | | |
|  | VTA | left | -2.0 | -17.5 | -7.0 | 139 | <0.001 | 6.35 |
|  | VTA | left | -0.5 | -20.5 | -17.5 |  | <0.001 | 5.10 |
|  | VTA | right | 8.5 | -16.0 | -11.5 |  | <0.001 | 4.52 |
| 3 | V | left | -0.5 | -59.5 | -1.0 | 126 | <0.001 | 4.07 |
| 4 | white matter |  | -9.5 | -38.5 | -31.0 | 17 | 0.002 | 3.08 |
| 5 | I-IV | right | 28.0 | -31.0 | -31.0 | 11 | 0.007 | 2.57 |
| 6 | VI | right | 35.5 | -34.0 | -35.5 | 3 | 0.029 | 1.94 |
| 7 | I-IV | right | 1.0 | -55.0 | -10.0 | 6 | 0.036 | 1.84 |
| 8 | white matter |  | -12.5 | -37.0 | -41.5 | 1 | 0.036 | 1.84 |
| 9 | V | left | -2.0 | -61.0 | -14.5 | 3 | 0.041 | 1.77 |
| 10 | Crus II | right | 14.5 | -74.5 | -35.5 | 3 | 0.042 | 1.76 |
| 11 | VI | right | 32.5 | -34.0 | -37.0 | 1 | 0.046 | 1.71 |
| *Figure 6D: First 3 no US post CS+ > no US post CS- reextinction, t-test, p < 0.05, uncorrected* | | | | | | | | |
| 1 | Extended cluster | left Crus I (2250), left Crus II (1542), left VI (1426), left VIIb (606), white matter (460), left I-IV (322), right I-IV (241), left IX (220), vermal IX (147), left DN (118), right V (79), left VIIIa (73), left VIIIb (67), left V (56), right DN (44), vermal VI (29), right IN (18), vermal VIIIa (15), left IN (14), vermal Crus I (7), vermal VIIIb (6), vermal X (6), right IX (4), left FN (4), right FN (2), right VI (1) | | | | | | |
|  | VI | left | -8.0 | -76.0 | -26.5 | 7757 | <0.001 | 6.40 |
|  | VI | left | -30.5 | -58.0 | -31.0 |  | <0.001 | 5.84 |
|  | Crus I | left | -15.5 | -79.0 | -25.0 |  | <0.001 | 5.76 |
| 2 | Extended cluster | left VTA (73), right VTA (69) | | | | | | |
|  | VTA | left | -5.0 | -16.0 | -13.0 | 142 | <0.001 | 5.04 |
|  | VTA | right | 4.0 | -14.5 | -13.0 |  | <0.001 | 5.03 |
| 3 | Extended cluster | right VI (905), right Crus I (585), right VIIb (498), right VIIIa (433), right X (161), white matter (135), right VIIIb (40), vermal VI (34), right Crus II (29), right V (11), right IX (1) | | | | | | |
|  | Crus I | right | 38.5 | -53.5 | -31.0 | 2832 | <0.001 | 4.58 |
|  | Crus I | right | 43.0 | -61.0 | -29.5 |  | <0.001 | 3.54 |
|  | VIIb | right | 14.5 | -74.5 | -47.5 |  | 0.001 | 3.51 |
| 4 | white matter |  | -23.0 | -47.5 | -43.0 | 48 | 0.008 | 2.51 |
| 5 | VIIIa | vermal | 7.0 | -64.0 | -38.5 | 21 | 0.017 | 2.20 |
| 6 | IX | right | 11.5 | -59.5 | -47.5 | 27 | 0.017 | 2.19 |
| 7 | white matter |  | -20.0 | -40.0 | -37.0 | 14 | 0.018 | 2.15 |
| 8 | VI | vermal | 4.0 | -70.0 | -16.0 | 13 | 0.025 | 2.01 |
| 9 | VIIIb | right | 11.5 | -49.0 | -61.0 | 5 | 0.028 | 1.96 |
| 10 | white matter |  | -15.5 | -34.0 | -40.0 | 4 | 0.032 | 1.90 |
| 11 | I-IV | left | -11.0 | -41.5 | -16.0 | 5 | 0.032 | 1.89 |
| 12 | I-IV | left | -8.0 | -50.5 | -10.0 | 3 | 0.036 | 1.83 |
| 13 | VI | left | -35.0 | -35.5 | -35.5 | 1 | 0.043 | 1.75 |
| 14 | VI | vermal | -0.5 | -68.5 | -26.5 | 1 | 0.045 | 1.72 |
| 15 | VI | vermal | 1.0 | -70.0 | -28.0 | 1 | 0.046 | 1.71 |
| 16 | Crus I | right | 23.5 | -73.0 | -29.5 | 1 | 0.049 | 1.68 |
| *Figure 7A: No US post CS+ x prediction error during extinction, t-test, p < 0.05, uncorrected* | | | | | | | | |
| 1 | Extended cluster | left Crus I (3530), left Crus II (2289), white matter (1583), left VI (1506), right Crus I (1298), right VI (798), left VIIb (606), left IX (536), left I-IV (254), vermal VIIIa (246), right I-IV (232), left DN (215), right DN (198), vermal IX (177), left VIIIb (173), right V (136), left X (127), vermal VI (126), right Crus II (85), left VIIIa (85), vermal VIIIb (69), left V (66), vermal X (60), vermal Crus II (55), vermal VIIb (41), right IX (20), left IN (18), right IN (16), right FN (8), left FN (7), vermal Crus I (6) | | | | | | |
|  | Crus I | left | -11.0 | -79.0 | -26.5 | 14566 | <0.001 | 6.39 |
|  | VI | left | -23.0 | -68.5 | -28.0 |  | <0.001 | 5.55 |
|  | VI | left | -33.5 | -50.5 | -32.5 |  | <0.001 | 5.20 |
| 2 | Extended cluster | right VTA (81), left VTA (71) | | | | | | |
|  | VTA | left | -5.0 | -17.5 | -13.0 | 152 | <0.001 | 4.45 |
|  | VTA | right | 5.5 | -17.5 | -14.5 |  | <0.001 | 3.77 |
| 3 | Extended cluster | right Crus II (410), right VIIb (296), right VIIIa (4) | | | | | | |
|  | Crus II | right | 29.5 | -82.0 | -49.0 | 710 | <0.001 | 4.04 |
|  | Crus II | right | 8.5 | -79.0 | -43.0 |  | <0.001 | 3.74 |
|  | VIIb | right | 20.5 | -71.5 | -52.0 |  | 0.001 | 3.42 |
| 4 | Extended cluster | right X (112), white matter (45), right VI (25), right VIIIa (19), right VIIIb (17), right VIIb (2) | | | | | | |
|  | VI | right | 31.0 | -41.5 | -41.5 | 220 | 0.001 | 3.40 |
|  | X | right | 20.5 | -37.0 | -49.0 |  | 0.006 | 2.61 |
|  | X | right | 22.0 | -32.5 | -40.0 |  | 0.027 | 1.98 |
| 5 | Extended cluster | right VIIb (107), right VIIIa (92), right Crus II (39), right VIIIb (8), white matter (4) | | | | | | |
|  | VIIb | right | 41.5 | -56.5 | -53.5 | 250 | 0.001 | 3.16 |
|  | VIIIa | right | 32.5 | -52.0 | -49.0 |  | 0.008 | 2.51 |
|  | VIIIb | right | 28.0 | -44.5 | -55.0 |  | 0.022 | 2.07 |
| 6 | Extended cluster | right V (31), left V (8) | | | | | | |
|  | V | right | 1.0 | -62.5 | -1.0 | 39 | 0.005 | 2.68 |
|  | V | right | 5.5 | -67.0 | -8.5 |  | 0.006 | 2.60 |
| 7 | Extended cluster | right I-IV (23) | | | | | | |
|  | I-IV | right | 5.5 | -49.0 | -1.0 | 23 | 0.006 | 2.60 |
|  | I-IV | right | 2.5 | -56.5 | 0.5 |  | 0.019 | 2.14 |
| 8 | I-IV | left | -5.0 | -49.0 | -1.0 | 7 | 0.008 | 2.49 |
| 9 | Crus II | right | 31.0 | -68.5 | -41.5 | 83 | 0.01 | 2.44 |
| 10 | Crus I | left | -50.0 | -70.0 | -40.0 | 12 | 0.014 | 2.28 |
| 11 | IX | right | 8.5 | -58.0 | -53.5 | 18 | 0.014 | 2.27 |
| 12 | V | left | -26.0 | -31.0 | -29.5 | 6 | 0.019 | 2.14 |
| 13 | white matter |  | 5.5 | -71.5 | -11.5 | 9 | 0.024 | 2.03 |
| 14 | VI | right | 38.5 | -38.5 | -29.5 | 5 | 0.028 | 1.95 |
| 15 | VI | right | 26.5 | -58.0 | -29.5 | 9 | 0.033 | 1.87 |
| 16 | Crus II | right | 46.0 | -47.5 | -46.0 | 2 | 0.034 | 1.86 |
| 17 | VIIIa | left | -32.0 | -47.5 | -56.5 | 1 | 0.038 | 1.82 |
| 18 | I-IV | left | -8.0 | -46.0 | -4.0 | 2 | 0.042 | 1.76 |
| 19 | VI | left | -38.0 | -37.0 | -31.0 | 1 | 0.042 | 1.76 |
| 20 | VI | right | 41.5 | -41.5 | -32.5 | 3 | 0.042 | 1.75 |
| *Figure 7B: No US post CS+ x prediction error during recall, t-test, p < 0.05, uncorrected* | | | | | | | | |
| 1 | Extended cluster | left Crus I (1637), left VI (1505), right VI (992), white matter (767), left Crus II (418), vermal VI (364), left VIIIb (322), vermal VIIIa (308), left X (221), left VIIIa (190), vermal IX (186), left IX (175), vermal VIIIb (154), right DN (151), right X (140), right Crus I (112), left I-IV (110), left DN (92), right I-IV (67), right Crus II (64), right V (62), vermal Crus II (62), vermal VIIb (55), left V (53), right VIIIb (49), right IX (41), vermal X (38), right VIIb (25), right VIIIa (18), left VIIb (10), left IN (10), vermal Crus I (4), right IN (1), right FN (1) | | | | | | |
|  | VI | left | -33.5 | -55.0 | -32.5 | 8404 | <0.001 | 7.24 |
|  | VI | left | -29.0 | -64.0 | -29.5 |  | <0.001 | 5.98 |
|  | VIIIb | vermal | -3.5 | -64.0 | -41.5 |  | <0.001 | 4.74 |
| 2 | Extended cluster | left VTA (51), right VTA (32) | | | | | | |
|  | VTA | left | -0.5 | -16.0 | -13.0 | 83 | <0.001 | 4.50 |
|  | VTA | right | 7.0 | -20.5 | -14.5 |  | 0.019 | 2.13 |
| 3 | Extended cluster | left Crus II (535), left VIIb (170), left VIIIa (16), left Crus I (9), white matter (1) | | | | | | |
|  | Crus II | left | -27.5 | -82.0 | -43.0 | 731 | <0.001 | 3.84 |
|  | Crus II | left | -23.0 | -76.0 | -46.0 |  | 0.006 | 2.60 |
|  | VIIIa | left | -18.5 | -70.0 | -55.0 |  | 0.015 | 2.23 |
| 4 | Extended cluster | right VIIb (278), right VIIIa (273), right VIIIb (135), right Crus II (125), white matter (93), right Crus I (2) | | | | | | |
|  | VIIIb | right | 29.5 | -46.0 | -53.5 | 906 | <0.001 | 3.83 |
|  | VIIb | right | 22.0 | -67.0 | -44.5 |  | 0.001 | 3.43 |
|  | VIIb | right | 40.0 | -44.5 | -50.5 |  | 0.002 | 3.04 |
| 5 | IX | left | -6.5 | -58.0 | -53.5 | 39 | 0.002 | 2.97 |
| 6 | Extended cluster | right I-IV (29), left V (6), right V (6), left I-IV (3) | | | | | | |
|  | I-IV | right | 4.0 | -52.0 | 0.5 | 44 | 0.003 | 2.84 |
|  | V | left | -0.5 | -61.0 | -1.0 |  | 0.01 | 2.43 |
| 7 | Crus II | left | -33.5 | -59.5 | -44.5 | 62 | 0.004 | 2.82 |
| 8 | Extended cluster | left Crus II (89), left VIIb (56) | | | | | | |
|  | Crus II | left | -45.5 | -52.0 | -50.5 | 145 | 0.004 | 2.77 |
|  | Crus II | left | -39.5 | -74.5 | -50.5 |  | 0.033 | 1.88 |
|  | Crus II | left | -42.5 | -67.0 | -50.5 |  | 0.038 | 1.81 |
| 9 | VIIb | left | -3.5 | -76.0 | -49.0 | 2 | 0.009 | 2.47 |
| 10 | Crus I | right | 34.0 | -79.0 | -22.0 | 21 | 0.012 | 2.33 |
| 11 | I-IV | right | 13.0 | -35.5 | -23.5 | 11 | 0.012 | 2.33 |
| 12 | Crus I | right | 43.0 | -55.0 | -34.0 | 34 | 0.014 | 2.28 |
| 13 | I-IV | right | 2.5 | -58.0 | -5.5 | 29 | 0.017 | 2.18 |
| 14 | VIIIb | right | 13.0 | -47.5 | -64.0 | 5 | 0.018 | 2.17 |
| 15 | Crus I | right | 43.0 | -64.0 | -34.0 | 21 | 0.018 | 2.15 |
| 16 | Crus I | right | 49.0 | -73.0 | -38.5 | 1 | 0.033 | 1.89 |
| 17 | VI | right | 37.0 | -43.0 | -25.0 | 1 | 0.036 | 1.83 |
| 18 | VI | right | 13.0 | -73.0 | -25.0 | 2 | 0.037 | 1.82 |
| 19 | VIIIa | left | -9.5 | -65.5 | -56.5 | 1 | 0.037 | 1.82 |
| 20 | Crus II | right | 41.5 | -68.5 | -52.0 | 5 | 0.038 | 1.81 |
| 21 | I-IV | left | -3.5 | -50.5 | -8.5 | 4 | 0.039 | 1.80 |
| 22 | VIIb | right | 34.0 | -65.5 | -56.5 | 1 | 0.041 | 1.77 |
| 23 | Crus I | left | -21.5 | -86.5 | -25.0 | 2 | 0.041 | 1.77 |
| 24 | IX | right | 5.5 | -58.0 | -55.0 | 1 | 0.043 | 1.75 |
| 25 | V | left | -6.5 | -58.0 | -13.0 | 1 | 0.047 | 1.70 |
| 26 | VIIIa | right | 17.5 | -64.0 | -58.0 | 1 | 0.048 | 1.69 |
| *Figure 7C: No US post CS+ x prediction error during reacquisition, t-test, p < 0.05, uncorrected* | | | | | | | | |
| 1 | Extended cluster | left Crus I (1123), left VI (925), left Crus II (863), left VIIb (381), white matter (53), left DN (5), left VIIIa (4) | | | | | | |
|  | Crus I | left | -27.5 | -68.5 | -31.0 | 3354 | <0.001 | 3.91 |
|  | VI | left | -30.5 | -56.5 | -32.5 |  | <0.001 | 3.77 |
|  | Crus I | left | -35.0 | -49.0 | -35.5 |  | 0.001 | 3.52 |
| 2 | Extended cluster | right VIIb (287), right Crus II (154), right VIIIa (154) | | | | | | |
|  | VIIIa | right | 32.5 | -61.0 | -53.5 | 595 | <0.001 | 3.70 |
|  | VIIb | right | 17.5 | -74.5 | -46.0 |  | <0.001 | 3.59 |
|  | VIIb | right | 31.0 | -71.5 | -55.0 |  | 0.002 | 3.01 |
| 3 | Extended cluster | right VTA (32), left VTA (9) | | | | | | |
|  | VTA | right | 5.5 | -13.0 | -11.5 | 41 | <0.001 | 3.61 |
|  | VTA | left | -0.5 | -20.5 | -17.5 |  | <0.001 | 3.60 |
| 4 | Extended cluster | right VI (270), right Crus I (124) | | | | | | |
|  | VI | right | 37.0 | -52.0 | -31.0 | 394 | 0.001 | 3.35 |
|  | VI | right | 32.5 | -64.0 | -25.0 |  | 0.039 | 1.80 |
| 5 | Extended cluster | left IX (175), vermal IX (77), right IX (70), left VIIIb (67), white matter (11), vermal VIIIa (4), vermal VIIIb (4), vermal X (2), left VIIIa (1) | | | | | | |
|  | IX | right | 2.5 | -58.0 | -56.5 | 411 | 0.001 | 3.29 |
|  | white matter |  | -9.5 | -58.0 | -41.5 |  | 0.005 | 2.71 |
|  | VIIIb | left | -9.5 | -62.5 | -52.0 |  | 0.008 | 2.51 |
| 6 | X | left | -24.5 | -34.0 | -46.0 | 32 | 0.001 | 3.23 |
| 7 | white matter |  | -24.5 | -41.5 | -40.0 | 77 | 0.003 | 2.91 |
| 8 | white matter |  | 26.5 | -43.0 | -43.0 | 52 | 0.005 | 2.68 |
| 9 | VTA | left | -5.0 | -13.0 | -11.5 | 11 | 0.012 | 2.33 |
| 10 | DN | left | -15.5 | -50.5 | -35.5 | 13 | 0.018 | 2.15 |
| 11 | IX | right | 5.5 | -49.0 | -59.5 | 3 | 0.024 | 2.02 |
| 12 | white matter |  | -9.5 | -40.0 | -38.5 | 4 | 0.027 | 1.98 |
| 13 | X | right | 20.5 | -35.5 | -46.0 | 9 | 0.028 | 1.96 |
| 14 | VTA | left | -3.5 | -16.0 | -14.5 | 3 | 0.031 | 1.91 |
| 15 | Crus I | right | 47.5 | -64.0 | -26.5 | 5 | 0.033 | 1.88 |
| 16 | VTA | left | -8.0 | -16.0 | -10.0 | 2 | 0.036 | 1.84 |
| 17 | Crus II | right | 44.5 | -71.5 | -44.5 | 3 | 0.038 | 1.81 |
| 18 | white matter |  | -9.5 | -38.5 | -31.0 | 2 | 0.039 | 1.80 |
| 19 | Crus II | right | 11.5 | -73.0 | -35.5 | 1 | 0.039 | 1.80 |
| 20 | DN | right | 17.5 | -59.5 | -34.0 | 2 | 0.039 | 1.80 |
| 21 | VIIIb | right | 10.0 | -44.5 | -61.0 | 4 | 0.039 | 1.79 |
| 22 | Crus I | left | -42.5 | -41.5 | -41.5 | 2 | 0.043 | 1.75 |
| 23 | VIIIb | right | 14.5 | -55.0 | -56.5 | 2 | 0.048 | 1.70 |
| 24 | VTA | right | 1.0 | -20.5 | -7.0 | 1 | 0.048 | 1.70 |
| *Figure 7D: No US post CS+ x prediction error during recall, t-test, p < 0.05, uncorrected* | | | | | | | | |
| 1 | Extended cluster | right VIIb (160), right Crus II (67), right VIIIa (31) | | | | | | |
|  | VIIb | right | 16.0 | -74.5 | -47.5 | 258 | 0.002 | 3.12 |
|  | VIIb | right | 31.0 | -71.5 | -53.5 |  | 0.014 | 2.28 |
|  | VIIIa | right | 32.5 | -61.0 | -53.5 |  | 0.017 | 2.19 |
| 2 | VIIIb | right | 11.5 | -46.0 | -61.0 | 41 | 0.003 | 2.92 |
| 3 | Extended cluster | left Crus II (216), left VIIb (179), white matter (46), left VIIIa (8), left Crus I (5) | | | | | | |
|  | VIIb | left | -32.0 | -62.5 | -49.0 | 454 | 0.005 | 2.70 |
|  | Crus II | left | -27.5 | -71.5 | -47.5 |  | 0.005 | 2.67 |
|  | VIIb | left | -15.5 | -73.0 | -49.0 |  | 0.007 | 2.54 |
| 4 | VTA | left | -0.5 | -20.5 | -17.5 | 12 | 0.005 | 2.66 |
| 5 | white matter |  | -24.5 | -41.5 | -40.0 | 63 | 0.006 | 2.61 |
| 6 | Extended cluster | left IX (26), right IX (26), left VIIIb (6), right VIIIb (3) | | | | | | |
|  | IX | left | -5.0 | -58.0 | -59.5 | 61 | 0.008 | 2.51 |
|  | IX | right | 2.5 | -58.0 | -56.5 |  | 0.014 | 2.26 |
|  | IX | right | 7.0 | -56.5 | -64.0 |  | 0.026 | 1.99 |
| 7 | X | left | -26.0 | -35.5 | -47.5 | 8 | 0.012 | 2.35 |
| 8 | Crus I | left | -38.0 | -62.5 | -31.0 | 37 | 0.016 | 2.22 |
| 9 | VI | left | -35.0 | -35.5 | -35.5 | 25 | 0.018 | 2.17 |
| 10 | VIIIb | right | 23.5 | -49.0 | -50.5 | 4 | 0.023 | 2.06 |
| 11 | VIIIa | vermal | -0.5 | -73.0 | -40.0 | 10 | 0.023 | 2.06 |
| 12 | I-IV | left | -5.0 | -55.0 | -16.0 | 4 | 0.023 | 2.06 |
| 13 | Crus II | left | -21.5 | -86.5 | -46.0 | 21 | 0.023 | 2.04 |
| 14 | I-IV | left | -9.5 | -37.0 | -22.0 | 11 | 0.024 | 2.03 |
| 15 | VI | left | -6.5 | -73.0 | -26.5 | 20 | 0.026 | 2.00 |
| 16 | Extended cluster | white matter (16), left DN (9), left VI (1) | | | | | | |
|  | white matter |  | -24.5 | -55.0 | -35.5 | 26 | 0.027 | 1.97 |
|  | white matter |  | -17.0 | -58.0 | -32.5 |  | 0.038 | 1.81 |
| 17 | VI | left | -32.0 | -55.0 | -34.0 | 21 | 0.028 | 1.96 |
| 18 | Crus II | right | 44.5 | -73.0 | -43.0 | 7 | 0.028 | 1.96 |
| 19 | white matter |  | -9.5 | -40.0 | -40.0 | 1 | 0.029 | 1.95 |
| 20 | Crus I | left | -15.5 | -80.5 | -25.0 | 9 | 0.03 | 1.92 |
| 21 | Crus I | left | -36.5 | -49.0 | -37.0 | 9 | 0.033 | 1.89 |
| 22 | white matter |  | -12.5 | -50.5 | -25.0 | 7 | 0.034 | 1.86 |
| 23 | VIIIb | left | -8.0 | -61.0 | -40.0 | 2 | 0.035 | 1.85 |
| 24 | Crus I | right | 40.0 | -52.0 | -31.0 | 10 | 0.036 | 1.83 |
| 25 | VIIIa | vermal | 5.5 | -67.0 | -38.5 | 5 | 0.037 | 1.83 |
| 26 | V | right | 4.0 | -58.0 | -26.5 | 3 | 0.038 | 1.80 |
| 27 | VTA | left | -5.0 | -13.0 | -11.5 | 1 | 0.04 | 1.78 |
| 28 | white matter |  | -11.0 | -68.5 | -32.5 | 9 | 0.04 | 1.78 |
| 29 | VTA | right | 4.0 | -14.5 | -13.0 | 1 | 0.042 | 1.76 |
| 30 | white matter |  | -15.5 | -40.0 | -40.0 | 3 | 0.042 | 1.76 |
| 31 | IX | vermal | -3.5 | -58.0 | -38.5 | 3 | 0.042 | 1.75 |
| 32 | VIIb | right | 40.0 | -68.5 | -55.0 | 1 | 0.046 | 1.72 |
| 33 | Crus II | right | 40.0 | -67.0 | -43.0 | 1 | 0.047 | 1.70 |
| 34 | Crus II | right | 35.5 | -71.5 | -47.5 | 1 | 0.049 | 1.68 |
