# Supplementary material for: Human cerebellum and ventral tegmental area interact during extinction of learned fear
Source: eLife. 2026 Jul 13;14:RP105399. doi: 10.7554/eLife.105399 (PMC13363218; doi:10.7554/eLife.105399)
Supplement: Supplementary file 12. — Clusters were identified in the cerebellar cortex and deep cerebellar nuclei (DCN). Up to three local maxima per cluster are reported, separated by at least 8 mm. Coordinates are given in MNI space (x, y, z). Cluster size is reported as number of voxels (voxel volume = 3.375 mm³). US: unconditioned stimulus; CS: conditioned stimulus; VTA: ventral tegmental area; DCN: deep cerebellar nuclei; DN: dentate nucleus; IN: interposed nucleus; FN: fastigial nucleus; MNI: Montreal Neurological Institute standard brain; t: t-statistic; punc: uncorrected p-value. [file elife-105399-supp12.docx]

## Supplementary fMRI results

### fMRI activation cluster tables

#### *fMRI activations related to PPI connectivity during unexpected US omissions with a VTA seed. Uncorrected.*

***Supplementary file 12:*** *fMRI psychophysiological interaction (PPI) activation clusters (p < 0.05, uncorrected) related to connectivity with the ventral tegmental area (VTA) during unexpected omission of the unconditioned stimulus (US) using a VTA seed (Figure 8). Clusters were identified in the cerebellar cortex and deep cerebellar nuclei (DCN). Up to three local maxima per cluster are reported, separated by at least 8 mm. Coordinates are given in MNI space (x, y, z). Cluster size is reported as number of voxels (voxel volume = 3.375 mm³). US: unconditioned stimulus; CS: conditioned stimulus; VTA: ventral tegmental area; DCN: deep cerebellar nuclei; DN: dentate nucleus; IN: interposed nucleus; FN: fastigial nucleus; MNI: Montreal Neurological Institute standard brain; t: t-statistic; p_unc_: uncorrected p-value.*

| **Index** | **Location (lobule, DCN)** | **Side** | **MNI coordinates/mm** | | | **Cluster size (number of voxels)** | **p_unc_** | **t** |
| --- | --- | --- | --- | --- | --- | --- | --- | --- |
|  |  |  | **x** | **y** | **z** |  |  |  |
| *Figure 8A: No US post CS+ x prediction error PPI positive connectivity with VTA during extinction, t-test, p < 0.05, uncorrected* | | | | | | | | |
| 1 | VIIIa | left | -24.5 | -68.5 | -59.5 | 107 | <0.001 | 4.14 |
| 2 | VI | left | -29.0 | -59.5 | -25.0 | 48 | 0.002 | 3.10 |
| 3 | VI | left | -35.0 | -35.5 | -34.0 | 12 | 0.002 | 3.04 |
| 4 | VIIIa | right | 10.0 | -70.0 | -55.0 | 10 | 0.007 | 2.58 |
| 5 | VIIb | left | -14.0 | -73.0 | -53.5 | 12 | 0.008 | 2.51 |
| 6 | VIIIa | vermal | -2.0 | -68.5 | -44.5 | 6 | 0.009 | 2.47 |
| 7 | VIIIb | right | 17.5 | -40.0 | -53.5 | 9 | 0.009 | 2.45 |
| 8 | V | right | 19.0 | -47.5 | -20.5 | 8 | 0.01 | 2.42 |
| 9 | VIIIb | right | 17.5 | -59.5 | -59.5 | 8 | 0.011 | 2.39 |
| 10 | V | right | 10.0 | -56.5 | -19.0 | 17 | 0.012 | 2.36 |
| 11 | VI | vermal | -3.5 | -67.0 | -23.5 | 6 | 0.013 | 2.32 |
| 12 | VI | right | 35.5 | -34.0 | -35.5 | 5 | 0.014 | 2.28 |
| 14 | Crus II | left | -30.5 | -79.0 | -50.5 | 2 | 0.019 | 2.13 |
| 15 | Crus I | left | -23.0 | -89.5 | -32.5 | 1 | 0.021 | 2.09 |
| 16 | Crus II | right | 16.0 | -83.5 | -35.5 | 1 | 0.021 | 2.09 |
| 17 | Crus I | right | 32.5 | -86.5 | -34.0 | 1 | 0.022 | 2.08 |
| 18 | Crus II | left | -47.0 | -53.5 | -49.0 | 3 | 0.022 | 2.08 |
| 19 | Crus II | vermal | 4.0 | -74.5 | -32.5 | 1 | 0.024 | 2.03 |
| 20 | Crus I | right | 29.5 | -88.0 | -34.0 | 1 | 0.025 | 2.02 |
| *Figure 8A: No US post CS+ x prediction error PPI negative connectivity with VTA during extinction, t-test, p < 0.05, uncorrected* | | | | | | | | |
| 1 | white matter |  | 29.5 | -65.5 | -44.5 | 84 | 0.001 | 3.36 |
| 2 | Extended cluster | white matter (216), left VI (12), left VIIIa (1) | | | | | | |
|  | white matter |  | -20.0 | -38.5 | -37.0 | 229 | 0.002 | 3.14 |
|  | white matter |  | -23.0 | -52.0 | -40.0 |  | 0.003 | 2.91 |
|  | white matter |  | -26.0 | -43.0 | -40.0 |  | 0.008 | 2.51 |
| 3 | Extended cluster | white matter (37), right IX (29), vermal X (15), right IN (10), vermal IX (8), vermal VIIIa (2) | | | | | | |
|  | IN | right | 7.0 | -58.0 | -31.0 | 101 | 0.002 | 3.12 |
|  | X | vermal | 5.5 | -49.0 | -34.0 |  | 0.007 | 2.57 |
| 4 | DN | left | -12.5 | -50.5 | -32.5 | 82 | 0.002 | 3.07 |
| 5 | Extended cluster | white matter (124), right X (4), right IX (1) | | | | | | |
|  | white matter |  | 11.5 | -41.5 | -40.0 | 129 | 0.002 | 3.04 |
|  | white matter |  | 23.5 | -52.0 | -43.0 |  | 0.011 | 2.39 |
| 6 | Extended cluster | white matter (127), left Crus II (38), left Crus I (15), left DN (9) | | | | | | |
|  | white matter |  | -17.0 | -70.0 | -40.0 | 189 | 0.002 | 2.99 |
|  | white matter |  | -18.5 | -61.0 | -38.5 |  | 0.006 | 2.60 |
| 7 | IX | right | 5.5 | -56.5 | -64.0 | 10 | 0.004 | 2.83 |
| 8 | V | right | 8.5 | -55.0 | -4.0 | 38 | 0.005 | 2.70 |
| 9 | Crus I | right | 47.5 | -67.0 | -34.0 | 32 | 0.006 | 2.60 |
| 10 | Crus II | right | 37.0 | -58.0 | -43.0 | 19 | 0.006 | 2.60 |
| 11 | Crus I | left | -36.5 | -58.0 | -31.0 | 34 | 0.006 | 2.60 |
| 12 | I-IV | left | -20.0 | -32.5 | -22.0 | 2 | 0.011 | 2.39 |
| 13 | Crus I | left | -26.0 | -79.0 | -26.5 | 10 | 0.011 | 2.37 |
| 14 | Crus I | right | 17.5 | -85.0 | -25.0 | 35 | 0.011 | 2.36 |
| 15 | VIIIb | vermal | 4.0 | -61.0 | -40.0 | 2 | 0.012 | 2.33 |
| 16 | Crus I | left | -14.0 | -86.5 | -23.5 | 23 | 0.013 | 2.31 |
| 17 | VIIIa | right | 34.0 | -49.0 | -46.0 | 8 | 0.014 | 2.28 |
| 18 | Crus II | left | -30.5 | -67.0 | -47.5 | 9 | 0.014 | 2.27 |
| 19 | I-IV | right | 19.0 | -34.0 | -19.0 | 1 | 0.015 | 2.25 |
| 20 | white matter |  | 23.5 | -62.5 | -40.0 | 13 | 0.015 | 2.24 |
| 21 | Crus II | left | -38.0 | -70.0 | -43.0 | 3 | 0.016 | 2.22 |
| 22 | VIIIb | right | 10.0 | -62.5 | -55.0 | 4 | 0.016 | 2.20 |
| 23 | Crus I | left | -42.5 | -61.0 | -37.0 | 4 | 0.017 | 2.20 |
| 24 | IX | right | 2.5 | -55.0 | -56.5 | 4 | 0.017 | 2.18 |
| 25 | Crus II | left | -3.5 | -88.0 | -29.5 | 1 | 0.018 | 2.17 |
| 26 | white matter |  | -3.5 | -55.0 | -29.5 | 1 | 0.018 | 2.16 |
| 27 | VIIIb | left | -18.5 | -53.5 | -50.5 | 5 | 0.019 | 2.14 |
| 28 | VI | right | 28.0 | -43.0 | -32.5 | 3 | 0.019 | 2.13 |
| 29 | VIIb | right | 22.0 | -77.5 | -55.0 | 1 | 0.02 | 2.11 |
| 30 | IX | right | 4.0 | -64.0 | -53.5 | 2 | 0.02 | 2.11 |
| 31 | Crus I | left | -45.5 | -59.5 | -25.0 | 6 | 0.02 | 2.11 |
| 32 | Crus II | right | 10.0 | -80.5 | -50.5 | 1 | 0.021 | 2.10 |
| 33 | VIIb | left | -5.0 | -74.5 | -50.5 | 1 | 0.022 | 2.06 |
| 34 | Crus I | left | -45.5 | -65.5 | -34.0 | 1 | 0.023 | 2.05 |
| 35 | Crus II | right | 13.0 | -82.0 | -50.5 | 1 | 0.024 | 2.04 |
| 36 | white matter |  | 29.5 | -50.5 | -41.5 | 1 | 0.024 | 2.04 |
| 37 | IX | vermal | 2.5 | -50.5 | -40.0 | 1 | 0.025 | 2.02 |
| *Figure 8A: No US post CS+ x prediction error PPI positive connectivity with VTA during recall, t-test, p <0.05, uncorrected* | | | | | | | | |
| 1 | Extended cluster | left VI (203), left Crus I (65), left V (52) | | | | | | |
|  | V | left | -21.5 | -50.5 | -20.5 | 320 | <0.001 | 4.07 |
|  | Crus I | left | -39.5 | -59.5 | -26.5 |  | <0.001 | 3.58 |
|  | VI | left | -32.0 | -58.0 | -23.5 |  | 0.001 | 3.21 |
| 2 | Extended cluster | left VIIIa (94), left VIIIb (69), left IX (63), vermal VIIIa (25), left VIIb (15), vermal VIIIb (5), white matter (2), left X (2) | | | | | | |
|  | VIIIa | left | -5.0 | -68.5 | -46.0 | 275 | 0.001 | 3.53 |
|  | VIIIb | left | -14.0 | -44.5 | -49.0 |  | 0.004 | 2.81 |
|  | VIIIa | left | -11.0 | -65.5 | -50.5 |  | 0.006 | 2.65 |
| 3 | Extended cluster | left I-IV (60), right I-IV (54) | | | | | | |
|  | I-IV | left | -0.5 | -43.0 | -14.5 | 114 | 0.001 | 3.41 |
|  | I-IV | right | 4.0 | -46.0 | -23.5 |  | 0.001 | 3.26 |
|  | I-IV | left | -6.5 | -43.0 | -23.5 |  | 0.013 | 2.31 |
| 4 | Extended cluster | right VI (134), right Crus I (67), white matter (11) | | | | | | |
|  | VI | right | 25.0 | -68.5 | -29.5 | 212 | 0.001 | 3.29 |
|  | Crus I | right | 34.0 | -68.5 | -28.0 |  | 0.003 | 2.90 |
|  | VI | right | 19.0 | -61.0 | -29.5 |  | 0.004 | 2.75 |
| 5 | I-IV | left | -15.5 | -37.0 | -25.0 | 69 | 0.001 | 3.28 |
| 6 | Extended cluster | vermal VI (104), left VI (28), right VI (17), left Crus II (12), vermal Crus II (12), vermal Crus I (5), right Crus I (3), left Crus I (1) | | | | | | |
|  | Crus II | vermal | -0.5 | -79.0 | -28.0 | 182 | 0.001 | 3.24 |
|  | VI | right | 7.0 | -71.5 | -20.5 |  | 0.004 | 2.78 |
|  | VI | left | -6.5 | -73.0 | -20.5 |  | 0.004 | 2.77 |
| 7 | Extended cluster | right IX (107), right VIIIa (70), vermal VIIIb (41), vermal VIIIa (39), vermal IX (36), right VIIIb (30), right VIIb (7), white matter (4) | | | | | | |
|  | VIIIa | right | 13.0 | -67.0 | -47.5 | 334 | 0.002 | 3.08 |
|  | VIIIb | vermal | 7.0 | -62.5 | -37.0 |  | 0.003 | 2.90 |
|  | IX | right | 11.5 | -56.5 | -49.0 |  | 0.003 | 2.89 |
| 8 | Extended cluster | right VI (198), right V (155), right I-IV (26) | | | | | | |
|  | V | right | 20.5 | -46.0 | -17.5 | 379 | 0.002 | 3.01 |
|  | VI | right | 29.5 | -55.0 | -28.0 |  | 0.003 | 2.94 |
|  | V | right | 7.0 | -56.5 | -7.0 |  | 0.003 | 2.92 |
| 9 | VIIb | right | 41.5 | -53.5 | -49.0 | 46 | 0.003 | 2.86 |
| 10 | IX | left | -2.0 | -55.0 | -46.0 | 76 | 0.005 | 2.68 |
| 11 | VI | left | -24.5 | -73.0 | -22.0 | 49 | 0.005 | 2.68 |
| 12 | Extended cluster | left Crus I (59), white matter (5) | | | | | | |
|  | Crus I | left | -39.5 | -76.0 | -34.0 | 64 | 0.006 | 2.64 |
| 13 | VI | left | -32.0 | -44.5 | -29.5 | 25 | 0.006 | 2.61 |
| 14 | Crus II | right | 22.0 | -86.5 | -41.5 | 17 | 0.006 | 2.59 |
| 15 | VIIIa | right | 31.0 | -44.5 | -49.0 | 14 | 0.007 | 2.59 |
| 16 | VI | left | -17.0 | -62.5 | -29.5 | 7 | 0.007 | 2.55 |
| 17 | VI | right | 34.0 | -41.5 | -28.0 | 24 | 0.007 | 2.54 |
| 18 | V | right | 4.0 | -64.0 | -7.0 | 31 | 0.008 | 2.50 |
| 19 | Crus II | left | -47.0 | -55.0 | -49.0 | 20 | 0.009 | 2.45 |
| 20 | Crus I | left | -33.5 | -74.5 | -25.0 | 26 | 0.01 | 2.40 |
| 21 | VIIIb | right | 17.5 | -56.5 | -55.0 | 12 | 0.01 | 2.40 |
| 22 | V | left | -9.5 | -50.5 | -8.5 | 10 | 0.012 | 2.35 |
| 23 | I-IV | left | -8.0 | -43.0 | -7.0 | 6 | 0.012 | 2.33 |
| 24 | VIIIb | left | -23.0 | -47.5 | -59.5 | 5 | 0.012 | 2.32 |
| 25 | X | left | -21.5 | -34.0 | -46.0 | 26 | 0.013 | 2.31 |
| 26 | Crus II | right | 28.0 | -80.5 | -40.0 | 8 | 0.014 | 2.27 |
| 27 | Crus I | right | 41.5 | -67.0 | -37.0 | 5 | 0.014 | 2.26 |
| 28 | Crus I | left | -20.0 | -83.5 | -29.5 | 8 | 0.016 | 2.22 |
| 29 | Crus I | left | -12.5 | -86.5 | -28.0 | 5 | 0.016 | 2.22 |
| 30 | Crus II | right | 38.5 | -82.0 | -40.0 | 1 | 0.016 | 2.22 |
| 31 | Crus II | left | -36.5 | -40.0 | -41.5 | 2 | 0.016 | 2.22 |
| 32 | VI | left | -6.5 | -67.0 | -10.0 | 11 | 0.016 | 2.21 |
| 33 | VI | right | 38.5 | -61.0 | -22.0 | 1 | 0.016 | 2.20 |
| 34 | VI | vermal | 1.0 | -64.0 | -20.5 | 4 | 0.016 | 2.20 |
| 35 | VIIIb | right | 20.5 | -43.0 | -47.5 | 6 | 0.017 | 2.19 |
| 36 | VI | left | -15.5 | -67.0 | -26.5 | 3 | 0.017 | 2.19 |
| 37 | Crus I | left | -33.5 | -70.0 | -32.5 | 4 | 0.017 | 2.19 |
| 38 | VIIb | left | -36.5 | -41.5 | -47.5 | 4 | 0.017 | 2.18 |
| 39 | VIIIa | left | -30.5 | -47.5 | -52.0 | 3 | 0.018 | 2.15 |
| 40 | X | left | -14.0 | -38.5 | -46.0 | 1 | 0.019 | 2.14 |
| 41 | I-IV | right | 7.0 | -47.5 | -7.0 | 3 | 0.02 | 2.12 |
| 42 | V | left | -11.0 | -59.5 | -19.0 | 2 | 0.022 | 2.08 |
| 43 | VI | left | -24.5 | -64.0 | -28.0 | 1 | 0.022 | 2.07 |
| 44 | VI | right | 35.5 | -59.5 | -22.0 | 1 | 0.023 | 2.05 |
| 45 | VI | right | 16.0 | -74.5 | -20.5 | 1 | 0.023 | 2.05 |
| 46 | VI | left | -36.5 | -41.5 | -28.0 | 1 | 0.023 | 2.05 |
| 47 | VIIIa | right | 22.0 | -61.0 | -59.5 | 1 | 0.023 | 2.05 |
| 48 | I-IV | right | 13.0 | -40.0 | -28.0 | 1 | 0.024 | 2.03 |
| 49 | white matter |  | -9.5 | -59.5 | -38.5 | 1 | 0.024 | 2.03 |
| 50 | Crus II | left | -36.5 | -80.5 | -43.0 | 1 | 0.024 | 2.02 |
| 51 | V | right | 10.0 | -61.0 | -19.0 | 1 | 0.025 | 2.02 |
| *Figure 8A: No US post CS+ x prediction error PPI negative connectivity with VTA during recall, t-test, p < 0.05, uncorrected* | | | | | | | | |
| 1 | white matter |  | -20.0 | -34.0 | -32.5 | 17 | 0.002 | 2.98 |
| 2 | white matter |  | -8.0 | -68.5 | -34.0 | 15 | 0.003 | 2.84 |
| 3 | VIIb | right | 40.0 | -64.0 | -53.5 | 24 | 0.004 | 2.83 |
| 4 | Crus II | right | 4.0 | -80.5 | -44.5 | 13 | 0.004 | 2.77 |
| 5 | Crus II | left | -8.0 | -91.0 | -35.5 | 3 | 0.011 | 2.40 |
| 6 | VIIb | left | -39.5 | -59.5 | -58.0 | 33 | 0.011 | 2.38 |
| 7 | white matter |  | 26.5 | -44.5 | -41.5 | 9 | 0.011 | 2.37 |
| 8 | Crus II | right | 8.5 | -89.5 | -34.0 | 2 | 0.012 | 2.34 |
| 9 | VI | left | -27.5 | -38.5 | -32.5 | 5 | 0.015 | 2.25 |
| 10 | Crus I | right | 28.0 | -83.5 | -26.5 | 1 | 0.02 | 2.11 |
| 11 | I-IV | right | 20.5 | -32.5 | -22.0 | 1 | 0.021 | 2.09 |
| 12 | Crus I | right | 44.5 | -77.5 | -31.0 | 2 | 0.022 | 2.08 |
| 13 | VIIb | right | 26.5 | -68.5 | -55.0 | 1 | 0.023 | 2.05 |
| 14 | white matter |  | -15.5 | -34.0 | -37.0 | 1 | 0.023 | 2.05 |
| 15 | Crus I | right | 44.5 | -59.5 | -29.5 | 1 | 0.024 | 2.03 |
| *Figure 8A: No US post CS+ x prediction error PPI positive connectivity with VTA during reacquisition, t-test, p < 0.05, uncorrected* | | | | | | | | |
| 1 | I-IV | left | -18.5 | -32.5 | -22.0 | 52 | <0.001 | 3.95 |
| 2 | VIIb | left | -5.0 | -67.0 | -32.5 | 82 | 0.001 | 3.26 |
| 3 | Extended cluster | right Crus II (108), right VIIb (9) | | | | | | |
|  | Crus II | right | 25.0 | -80.5 | -44.5 | 117 | 0.001 | 3.16 |
|  | Crus II | right | 28.0 | -71.5 | -47.5 |  | 0.014 | 2.29 |
| 4 | Extended cluster | left Crus I (96) | | | | | | |
|  | Crus I | left | -45.5 | -58.0 | -35.5 | 96 | 0.002 | 3.06 |
|  | Crus I | left | -39.5 | -65.5 | -26.5 |  | 0.015 | 2.25 |
| 5 | VIIb | left | -39.5 | -59.5 | -55.0 | 25 | 0.003 | 2.88 |
| 6 | white matter |  | 7.0 | -62.5 | -28.0 | 34 | 0.003 | 2.88 |
| 7 | Crus II | right | 7.0 | -82.0 | -46.0 | 9 | 0.004 | 2.75 |
| 8 | VI | left | -21.5 | -62.5 | -28.0 | 93 | 0.005 | 2.71 |
| 9 | Extended cluster | right IX (20), left IX (12), left VIIIb (1) | | | | | | |
|  | IX | right | 2.5 | -62.5 | -56.5 | 33 | 0.005 | 2.68 |
|  | IX | left | -6.5 | -59.5 | -58.0 |  | 0.014 | 2.29 |
| 10 | V | right | 16.0 | -47.5 | -23.5 | 86 | 0.005 | 2.67 |
| 11 | I-IV | left | -11.0 | -41.5 | -10.0 | 15 | 0.006 | 2.65 |
| 12 | VI | right | 34.0 | -46.0 | -34.0 | 31 | 0.007 | 2.58 |
| 13 | I-IV | right | 17.5 | -32.5 | -20.5 | 25 | 0.007 | 2.57 |
| 14 | white matter |  | 17.5 | -47.5 | -41.5 | 11 | 0.007 | 2.56 |
| 15 | VIIIa | right | 26.5 | -58.0 | -61.0 | 6 | 0.007 | 2.54 |
| 16 | VIIIa | right | 11.5 | -70.0 | -56.5 | 3 | 0.009 | 2.48 |
| 17 | white matter |  | 31.0 | -61.0 | -43.0 | 9 | 0.009 | 2.47 |
| 18 | white matter |  | 25.0 | -67.0 | -41.5 | 37 | 0.009 | 2.46 |
| 19 | Crus I | right | 41.5 | -70.0 | -26.5 | 8 | 0.011 | 2.40 |
| 20 | white matter |  | 41.5 | -55.0 | -38.5 | 31 | 0.011 | 2.37 |
| 21 | VI | vermal | 2.5 | -77.5 | -16.0 | 3 | 0.012 | 2.34 |
| 22 | VIIIb | right | 25.0 | -52.0 | -58.0 | 5 | 0.013 | 2.32 |
| 23 | I-IV | right | 25.0 | -32.5 | -34.0 | 10 | 0.013 | 2.32 |
| 24 | VI | right | 19.0 | -65.5 | -28.0 | 4 | 0.014 | 2.28 |
| 25 | I-IV | right | 2.5 | -43.0 | -22.0 | 16 | 0.014 | 2.27 |
| 26 | I-IV | right | 11.5 | -43.0 | -16.0 | 14 | 0.015 | 2.25 |
| 27 | VI | right | 31.0 | -61.0 | -22.0 | 2 | 0.016 | 2.23 |
| 28 | Crus II | left | -20.0 | -82.0 | -50.5 | 6 | 0.016 | 2.22 |
| 29 | VI | right | 28.0 | -49.0 | -20.5 | 4 | 0.016 | 2.22 |
| 30 | Crus I | right | 40.0 | -53.5 | -29.5 | 5 | 0.016 | 2.21 |
| 31 | Crus II | left | -45.5 | -65.5 | -52.0 | 2 | 0.016 | 2.21 |
| 32 | VI | right | 35.5 | -56.5 | -23.5 | 6 | 0.016 | 2.21 |
| 33 | Crus II | left | -5.0 | -83.5 | -43.0 | 4 | 0.018 | 2.17 |
| 34 | Crus II | left | -33.5 | -82.0 | -47.5 | 1 | 0.018 | 2.16 |
| 35 | I-IV | right | 11.5 | -46.0 | -10.0 | 8 | 0.018 | 2.15 |
| 36 | VI | left | -32.0 | -67.0 | -26.5 | 10 | 0.019 | 2.15 |
| 37 | VI | left | -27.5 | -53.5 | -32.5 | 1 | 0.02 | 2.12 |
| 38 | VIIb | right | 20.5 | -70.0 | -52.0 | 1 | 0.022 | 2.07 |
| 39 | VIIb | left | -38.0 | -47.5 | -50.5 | 1 | 0.023 | 2.05 |
| 40 | VIIIb | right | 28.0 | -38.5 | -50.5 | 1 | 0.023 | 2.05 |
| 41 | VI | right | 29.5 | -74.5 | -20.5 | 1 | 0.023 | 2.05 |
| 42 | Crus II | left | -14.0 | -80.5 | -49.0 | 1 | 0.023 | 2.05 |
| 43 | Crus I | left | -50.0 | -61.0 | -26.5 | 1 | 0.023 | 2.05 |
| 44 | DN | left | -14.0 | -55.0 | -32.5 | 1 | 0.024 | 2.03 |
| 45 | VI | left | -35.0 | -53.5 | -28.0 | 1 | 0.024 | 2.03 |
| 46 | I-IV | right | 14.5 | -38.5 | -25.0 | 1 | 0.025 | 2.02 |
| *Figure 8A: No US post CS+ x prediction error PPI negative connectivity with VTA during reacquisition, t-test, p <0.05, uncorrected* | | | | | | | | |
| 1 | white matter |  | -11.0 | -47.5 | -23.5 | 52 | 0.001 | 3.46 |
| 2 | I-IV | right | 4.0 | -52.0 | -13.0 | 27 | 0.001 | 3.15 |
| 3 | Crus I | right | 43.0 | -47.5 | -28.0 | 5 | 0.002 | 3.04 |
| 4 | VIIb | right | 44.5 | -52.0 | -49.0 | 15 | 0.002 | 2.98 |
| 5 | VIIIb | left | -8.0 | -44.5 | -58.0 | 27 | 0.002 | 2.98 |
| 6 | Extended cluster | white matter (66), left Crus I (1), left Crus II (1) | | | | | | |
|  | white matter |  | -30.5 | -58.0 | -40.0 | 68 | 0.002 | 2.97 |
|  | white matter |  | -21.5 | -50.5 | -37.0 |  | 0.004 | 2.80 |
| 7 | white matter |  | 8.5 | -46.0 | -31.0 | 12 | 0.003 | 2.91 |
| 8 | VI | right | 34.0 | -40.0 | -26.5 | 7 | 0.009 | 2.48 |
| 9 | Crus II | right | 11.5 | -83.5 | -40.0 | 12 | 0.009 | 2.47 |
| 10 | Crus II | left | -12.5 | -85.0 | -40.0 | 9 | 0.009 | 2.46 |
| 11 | white matter |  | -15.5 | -40.0 | -31.0 | 20 | 0.012 | 2.35 |
| 12 | V | left | -21.5 | -37.0 | -29.5 | 9 | 0.014 | 2.27 |
| 13 | VIIIb | right | 16.0 | -49.0 | -52.0 | 12 | 0.014 | 2.26 |
| 14 | VIIb | right | 38.5 | -44.5 | -44.5 | 5 | 0.015 | 2.25 |
| 15 | VIIIa | right | 8.5 | -70.0 | -50.5 | 2 | 0.016 | 2.20 |
| 16 | Crus I | left | -48.5 | -47.5 | -40.0 | 1 | 0.019 | 2.13 |
| 17 | I-IV | left | -2.0 | -46.0 | -16.0 | 1 | 0.023 | 2.04 |
| *Figure 8A: No US post CS+ x prediction error PPI positive connectivity with VTA during reextinction, t-test, p < 0.05, uncorrected* | | | | | | | | |
| 1 | white matter |  | 22.0 | -32.5 | -34.0 | 20 | 0.002 | 2.97 |
| 2 | VIIIa | right | 11.5 | -70.0 | -56.5 | 11 | 0.003 | 2.87 |
| 3 | I-IV | left | -18.5 | -32.5 | -23.5 | 17 | 0.004 | 2.78 |
| 4 | VIIIb | left | -18.5 | -46.0 | -61.0 | 21 | 0.007 | 2.57 |
| 5 | Extended cluster | left VIIb (28), left VIIIa (11) | | | | | | |
|  | VIIb | left | -38.0 | -53.5 | -58.0 | 39 | 0.008 | 2.53 |
|  | VIIb | left | -38.0 | -61.0 | -53.5 |  | 0.017 | 2.20 |
|  | VIIIa | left | -29.0 | -49.0 | -58.0 |  | 0.018 | 2.16 |
| 6 | VIIIb | right | 11.5 | -50.5 | -61.0 | 9 | 0.008 | 2.52 |
| 7 | I-IV | right | 2.5 | -43.0 | -22.0 | 21 | 0.008 | 2.48 |
| 8 | VI | left | -23.0 | -59.5 | -29.5 | 26 | 0.009 | 2.48 |
| 9 | X | left | -15.5 | -37.0 | -49.0 | 3 | 0.011 | 2.37 |
| 10 | VIIIa | left | -29.0 | -40.0 | -50.5 | 11 | 0.012 | 2.34 |
| 11 | VIIb | right | 41.5 | -59.5 | -55.0 | 17 | 0.012 | 2.33 |
| 12 | Crus I | left | -50.0 | -68.5 | -41.5 | 2 | 0.014 | 2.28 |
| 13 | Crus I | left | -47.0 | -58.0 | -34.0 | 8 | 0.014 | 2.27 |
| 14 | VI | left | -36.5 | -53.5 | -28.0 | 9 | 0.014 | 2.26 |
| 15 | VIIIb | right | 14.5 | -56.5 | -61.0 | 7 | 0.016 | 2.21 |
| 16 | VIIIb | right | 23.5 | -50.5 | -58.0 | 5 | 0.016 | 2.21 |
| 17 | IX | left | -6.5 | -62.5 | -56.5 | 4 | 0.017 | 2.18 |
| 18 | Crus II | left | -6.5 | -85.0 | -43.0 | 3 | 0.019 | 2.14 |
| 19 | VIIb | left | -32.0 | -62.5 | -52.0 | 3 | 0.019 | 2.13 |
| 20 | VIIIb | right | 26.5 | -40.0 | -52.0 | 1 | 0.02 | 2.12 |
| 21 | Crus II | left | -8.0 | -91.0 | -35.5 | 2 | 0.02 | 2.11 |
| 22 | I-IV | right | 11.5 | -40.0 | -10.0 | 1 | 0.021 | 2.08 |
| 23 | Crus I | right | 50.5 | -62.5 | -32.5 | 3 | 0.022 | 2.07 |
| 24 | VIIIa | left | -32.0 | -43.0 | -52.0 | 1 | 0.023 | 2.05 |
| 25 | I-IV | right | 11.5 | -38.5 | -13.0 | 1 | 0.024 | 2.04 |
| 26 | VI | right | 37.0 | -58.0 | -23.5 | 2 | 0.024 | 2.03 |
| 27 | X | left | -24.5 | -34.0 | -41.5 | 1 | 0.024 | 2.03 |
| 28 | IX | left | -6.5 | -46.0 | -50.5 | 1 | 0.024 | 2.02 |
| *Figure 8A: No US post CS+ x prediction error PPI negative connectivity with VTA during reextinction, t-test, p < 0.05, uncorrected* | | | | | | | | |
| 1 | I-IV | right | 4.0 | -50.5 | -14.5 | 38 | 0.001 | 3.33 |
| 2 | VIIIb | left | -9.5 | -43.0 | -58.0 | 18 | 0.001 | 3.20 |
| 3 | white matter |  | -12.5 | -47.5 | -26.5 | 31 | 0.002 | 3.03 |
| 4 | Extended cluster | left V (20), white matter (9), left I-IV (9) | | | | | | |
|  | white matter |  | -18.5 | -31.0 | -31.0 | 38 | 0.003 | 2.93 |
|  | V | left | -26.0 | -35.5 | -28.0 |  | 0.016 | 2.22 |
| 5 | V | right | 31.0 | -38.5 | -25.0 | 24 | 0.003 | 2.86 |
| 6 | VI | left | -35.0 | -38.5 | -32.5 | 42 | 0.003 | 2.86 |
| 7 | Crus II | right | 7.0 | -80.5 | -41.5 | 29 | 0.004 | 2.79 |
| 8 | Extended cluster | right IX (116), right VIIIb (90), white matter (22), right VIIIa (17) | | | | | | |
|  | IX | right | 14.5 | -55.0 | -50.5 | 245 | 0.004 | 2.76 |
|  | IX | right | 8.5 | -56.5 | -44.5 |  | 0.005 | 2.69 |
|  | VIIIb | right | 17.5 | -44.5 | -50.5 |  | 0.016 | 2.22 |
| 9 | Extended cluster | white matter (187), left Crus II (25), left Crus I (17), left VIIIa (9), left VI (4), left DN (1) | | | | | | |
|  | white matter |  | -30.5 | -59.5 | -40.0 | 243 | 0.005 | 2.72 |
|  | white matter |  | -24.5 | -55.0 | -37.0 |  | 0.006 | 2.60 |
|  | VIIIa | left | -29.0 | -52.0 | -46.0 |  | 0.014 | 2.26 |
| 10 | V | right | 32.5 | -34.0 | -34.0 | 6 | 0.005 | 2.67 |
| 11 | white matter |  | -12.5 | -40.0 | -31.0 | 92 | 0.007 | 2.55 |
| 12 | I-IV | left | -5.0 | -49.0 | -13.0 | 19 | 0.008 | 2.53 |
| 13 | VI | vermal | 1.0 | -77.5 | -25.0 | 4 | 0.008 | 2.51 |
| 14 | VIIb | right | 11.5 | -71.5 | -49.0 | 29 | 0.009 | 2.48 |
| 15 | VIIIa | left | -8.0 | -70.0 | -53.5 | 24 | 0.009 | 2.44 |
| 16 | white matter |  | -14.0 | -53.5 | -40.0 | 15 | 0.011 | 2.38 |
| 17 | Crus I | right | 32.5 | -77.5 | -37.0 | 11 | 0.011 | 2.36 |
| 18 | VIIIb | left | -18.5 | -44.5 | -52.0 | 11 | 0.012 | 2.35 |
| 19 | Crus II | right | 14.5 | -85.0 | -37.0 | 7 | 0.013 | 2.31 |
| 20 | Crus I | left | -17.0 | -82.0 | -25.0 | 5 | 0.014 | 2.26 |
| 21 | Crus I | left | -48.5 | -47.5 | -40.0 | 1 | 0.014 | 2.26 |
| 22 | IX | left | -5.0 | -52.0 | -41.5 | 5 | 0.015 | 2.24 |
| 23 | X | vermal | -2.0 | -50.5 | -32.5 | 5 | 0.016 | 2.22 |
| 24 | Crus I | right | 44.5 | -65.5 | -43.0 | 2 | 0.019 | 2.14 |
| 25 | V | left | -23.0 | -46.0 | -17.5 | 1 | 0.02 | 2.12 |
| 26 | Crus II | left | -14.0 | -85.0 | -40.0 | 6 | 0.02 | 2.11 |
| 27 | IX | left | -5.0 | -49.0 | -37.0 | 1 | 0.021 | 2.10 |
| 28 | Crus I | left | -11.0 | -71.5 | -31.0 | 4 | 0.021 | 2.09 |
| 29 | white matter |  | -27.5 | -44.5 | -35.5 | 1 | 0.023 | 2.06 |
| 30 | Crus I | right | 46.0 | -61.0 | -43.0 | 1 | 0.023 | 2.04 |
| 31 | Crus II | right | 40.0 | -44.5 | -44.5 | 1 | 0.023 | 2.04 |
| *Figure 8B: No US post CS+ x prediction error PPI positive connectivity with VTA during extinction, recall, reacquisition and reextinction, t-test, p < 0.05, uncorrected* | | | | | | | | |
| 1 | Extended cluster | left Crus I (158), left VI (142), left V (14) | | | | | | |
|  | VI | left | -32.0 | -58.0 | -25.0 | 314 | 0.001 | 3.53 |
|  | VI | left | -21.5 | -52.0 | -19.0 |  | 0.002 | 2.99 |
|  | Crus I | left | -38.0 | -65.5 | -32.5 |  | 0.003 | 2.90 |
| 2 | I-IV | left | -14.0 | -37.0 | -25.0 | 105 | 0.001 | 3.30 |
| 3 | Extended cluster | left X (23), left VIIIb (11), left VIIIa (6) | | | | | | |
|  | X | left | -21.5 | -34.0 | -46.0 | 40 | 0.001 | 3.25 |
|  | VIIIa | left | -29.0 | -40.0 | -50.5 |  | 0.016 | 2.21 |
| 4 | Extended cluster | right V (225), right VI (32), white matter (21), right I-IV (16) | | | | | | |
|  | V | right | 17.5 | -47.5 | -22.0 | 294 | 0.001 | 3.19 |
|  | V | right | 13.0 | -53.5 | -19.0 |  | 0.003 | 2.94 |
| 5 | Extended cluster | left I-IV (33), right I-IV (32) | | | | | | |
|  | I-IV | left | -5.0 | -43.0 | -22.0 | 65 | 0.002 | 3.13 |
|  | I-IV | right | 4.0 | -43.0 | -20.5 |  | 0.004 | 2.76 |
| 6 | VIIIb | left | -24.5 | -46.0 | -58.0 | 31 | 0.002 | 3.08 |
| 7 | VIIIb | right | 19.0 | -59.5 | -59.5 | 53 | 0.002 | 3.03 |
| 8 | VIIb | left | -36.5 | -41.5 | -47.5 | 24 | 0.002 | 3.01 |
| 9 | VI | left | -23.0 | -61.0 | -28.0 | 151 | 0.003 | 2.85 |
| 10 | VIIIb | right | 25.0 | -52.0 | -58.0 | 13 | 0.005 | 2.69 |
| 11 | Crus II | vermal | -2.0 | -79.0 | -29.5 | 16 | 0.005 | 2.68 |
| 12 | white matter |  | 41.5 | -53.5 | -38.5 | 29 | 0.005 | 2.67 |
| 13 | VIIIb | right | 13.0 | -40.0 | -52.0 | 3 | 0.005 | 2.67 |
| 14 | V | right | 2.5 | -61.0 | -23.5 | 36 | 0.006 | 2.65 |
| 15 | VI | left | -6.5 | -73.0 | -22.0 | 25 | 0.006 | 2.62 |
| 16 | VI | right | 8.5 | -73.0 | -22.0 | 23 | 0.007 | 2.59 |
| 17 | VIIb | left | -26.0 | -68.5 | -55.0 | 8 | 0.007 | 2.57 |
| 18 | Extended cluster | right Crus I (33), right VI (27) | | | | | | |
|  | Crus I | right | 32.5 | -67.0 | -32.5 | 60 | 0.007 | 2.55 |
|  | VI | right | 22.0 | -62.5 | -29.5 |  | 0.014 | 2.26 |
| 19 | VIIIb | left | -15.5 | -38.5 | -50.5 | 4 | 0.009 | 2.47 |
| 20 | VI | right | 34.0 | -56.5 | -23.5 | 22 | 0.009 | 2.45 |
| 21 | V | right | 10.0 | -61.0 | -19.0 | 11 | 0.009 | 2.45 |
| 22 | Crus II | left | -21.5 | -79.0 | -52.0 | 6 | 0.01 | 2.41 |
| 23 | Crus II | left | -29.0 | -79.0 | -52.0 | 21 | 0.01 | 2.41 |
| 24 | V | right | 2.5 | -65.5 | -7.0 | 20 | 0.01 | 2.41 |
| 25 | I-IV | left | -8.0 | -43.0 | -8.5 | 12 | 0.01 | 2.41 |
| 26 | Crus I | left | -45.5 | -58.0 | -32.5 | 9 | 0.01 | 2.41 |
| 27 | VI | right | 28.0 | -67.0 | -23.5 | 50 | 0.013 | 2.32 |
| 28 | VIIIa | right | 10.0 | -70.0 | -56.5 | 3 | 0.013 | 2.30 |
| 29 | VIIIa | left | -8.0 | -68.5 | -44.5 | 6 | 0.014 | 2.27 |
| 30 | VIIIb | right | 23.5 | -46.0 | -58.0 | 3 | 0.015 | 2.25 |
| 31 | VI | left | -9.5 | -67.0 | -26.5 | 5 | 0.015 | 2.24 |
| 32 | VI | left | -33.5 | -43.0 | -25.0 | 6 | 0.015 | 2.23 |
| 33 | IX | right | 2.5 | -55.0 | -47.5 | 5 | 0.017 | 2.20 |
| 34 | VIIIa | left | -30.5 | -47.5 | -52.0 | 3 | 0.017 | 2.18 |
| 35 | I-IV | right | 14.5 | -40.0 | -28.0 | 3 | 0.018 | 2.15 |
| 36 | VIIIa | vermal | 5.5 | -70.0 | -44.5 | 4 | 0.019 | 2.15 |
| 37 | Crus II | left | -33.5 | -80.5 | -47.5 | 2 | 0.022 | 2.08 |
| 38 | Crus II | left | -15.5 | -80.5 | -50.5 | 1 | 0.022 | 2.08 |
| 39 | IX | right | 1.0 | -62.5 | -55.0 | 2 | 0.022 | 2.07 |
| 40 | Crus I | left | -32.0 | -79.0 | -31.0 | 1 | 0.022 | 2.07 |
| 41 | Crus II | left | -17.0 | -85.0 | -49.0 | 1 | 0.023 | 2.04 |
| 42 | Crus II | right | 26.5 | -80.5 | -41.5 | 1 | 0.024 | 2.03 |
| 43 | X | right | 23.5 | -34.0 | -47.5 | 1 | 0.024 | 2.03 |
| 44 | Crus II | left | -47.0 | -56.5 | -46.0 | 1 | 0.025 | 2.02 |
| *Figure 8B: No US post CS+ x prediction error PPI negative connectivity with VTA during extinction, recall, reacquisition and reextinction, t-test, p < 0.05, uncorrected* | | | | | | | | |
| 1 | Extended cluster | white matter (515), left V (72), left IX (25), left I-IV (14), left DN (12), left VI (10), vermal X (7), left Crus I (6), left Crus II (4), left X (2) | | | | | | |
|  | white matter |  | -18.5 | -31.0 | -32.5 | 667 | 0.001 | 3.24 |
|  | white matter |  | -24.5 | -53.5 | -37.0 |  | 0.002 | 3.12 |
|  | V | left | -26.0 | -37.0 | -31.0 |  | 0.002 | 3.00 |
| 2 | white matter |  | -9.5 | -70.0 | -34.0 | 21 | 0.004 | 2.77 |
| 3 | I-IV | right | 4.0 | -52.0 | -13.0 | 16 | 0.006 | 2.62 |
| 4 | white matter |  | 10.0 | -70.0 | -35.5 | 6 | 0.006 | 2.62 |
| 5 | Extended cluster | white matter (35), right DN (2) | | | | | | |
|  | white matter |  | 23.5 | -52.0 | -37.0 | 37 | 0.006 | 2.61 |
|  | white matter |  | 16.0 | -49.0 | -35.5 |  | 0.017 | 2.18 |
| 6 | white matter |  | 7.0 | -46.0 | -32.5 | 10 | 0.009 | 2.48 |
| 7 | VIIIa | right | 8.5 | -70.0 | -50.5 | 9 | 0.009 | 2.47 |
| 8 | VIIIa | left | -9.5 | -68.5 | -55.0 | 4 | 0.009 | 2.44 |
| 9 | Crus II | right | 13.0 | -82.0 | -50.5 | 2 | 0.011 | 2.38 |
| 10 | Crus II | right | 8.5 | -80.5 | -40.0 | 9 | 0.013 | 2.32 |
| 11 | Crus II | left | -21.5 | -80.5 | -43.0 | 5 | 0.013 | 2.30 |
| 12 | VIIIb | left | -8.0 | -46.0 | -59.5 | 12 | 0.013 | 2.30 |
| 13 | white matter |  | 2.5 | -59.5 | -29.5 | 5 | 0.013 | 2.29 |
| 14 | Crus I | right | 41.5 | -46.0 | -41.5 | 5 | 0.015 | 2.26 |
| 15 | white matter |  | -11.0 | -46.0 | -25.0 | 3 | 0.016 | 2.21 |
| 16 | IX | right | 5.5 | -50.5 | -62.5 | 3 | 0.017 | 2.19 |
| 17 | white matter |  | 34.0 | -46.0 | -43.0 | 4 | 0.018 | 2.15 |
| 18 | VIIIb | right | 13.0 | -61.0 | -55.0 | 1 | 0.019 | 2.14 |
| 19 | VIIIb | right | 26.5 | -43.0 | -44.5 | 2 | 0.02 | 2.12 |
| 20 | Crus II | left | -12.5 | -85.0 | -40.0 | 2 | 0.021 | 2.10 |
