# Supplementary material for: Human cerebellum and ventral tegmental area interact during extinction of learned fear
Source: eLife. 2026 Jul 13;14:RP105399. doi: 10.7554/eLife.105399 (PMC13363218; doi:10.7554/eLife.105399)
Supplement: Peer review history

# Editor report: eLife Assessment

**DOI:** 10.7554/eLife.105399.3.sa0

**Authors:** Andre F Marquand

**Affiliations:** Radboud University Nijmegen Netherlands

**Keywords:** Important, Solid

This **important** study provides insights into the role of the cerebellum in fear conditioning, addressing a key gap in the literature. The evidence presented in support of the conclusions is **solid**. This work will be of interest to both the extinction learning and cerebellar research communities.

# Referee report: Reviewer #1 (Public review)

**DOI:** 10.7554/eLife.105399.3.sa1

Nio and colleagues address an important question about how the cerebellum and ventral tegmental area (VTA) contribute to extinction learning of conditioned fear associations. This work tackles a critical gap in the existing literature and provides new insights into this question in humans through the use of high-field neuroimaging with robust methodology. The presented results are novel and will broadly interest both the extinction learning and cerebellar research communities. As such, this is a very timely and important contribution.

Strengths:

The core finding - coupling of cerebellum and VTA as a reward-like prediction errors during fear extinction - is novel and addresses a genuine gap in the literature. Also the paradigm spanning several sessions, a well-powered sample, 7T imaging and complementary analytical approaches to target the question is commendable.

Weaknesses:

The authors have satisfactorily addressed the concerns raised in the previous version of the manuscript. Several results, as well as conclusions drawn from them, still rest on trend-level evidence, although the revised presentation of the results now provides a more balanced interpretation of these findings.

# Author comment: Author response

**DOI:** 10.7554/eLife.105399.3.sa2

**Authors:** Enzo Nio, Patrick Pais Pereira, Nicolas Diekmann, Mykola Petrenko, Alice Doubliez, Thomas Michael Ernst, Giorgi Batsikadze, Stefan Maderwald, Cornelius Deuschl, Metin Üngör, Sen Cheng, Christian Josef Merz, Harald H Quick, Dagmar Timmann

**Affiliations:** Department of Neurology and Center for Translational Neuro- and Behavioral Sciences (C-TNBS), Essen University Hospital, University of Duisburg-Essen Essen Germany; Department of Neurology and Center for Translational Neuro- and Behavioral Sciences (C-TNBS), Essen University Hospital, University of Duisburg-Essen Essen Germany; Ruhr University Bochum Bochum Germany; Department of Neurology and Center for Translational Neuro- and Behavioral Sciences (C-TNBS), Essen University Hospital, University of Duisburg-Essen Essen Germany; Department of Neurology and Center for Translational Neuro- and Behavioral Sciences (C-TNBS), Essen University Hospital, University of Duisburg-Essen Essen Germany; University Clinic Essen Essen Germany; University of Duisburg-Essen Essen Germany; University of Duisburg-Essen Essen Germany; University of Duisburg-Essen Essen Germany; Philipps-University Marburg Marburg Germany; Ruhr University Bochum Bochum Germany; Ruhr University Bochum Bochum Germany; University of Duisburg-Essen Essen Germany; Essen University Hospital Essen Germany

The following is the authors’ response to the original reviews.

**Public Reviews:**

**Reviewer #1 (Public review):**

Nio and colleagues address an important question about how the cerebellum and ventral tegmental area (VTA) contribute to the extinction learning of conditioned fear associations. This work tackles a critical gap in the existing literature and provides new insights into this question in humans through the use of high-field neuroimaging with robust methodology. The presented results are novel and will broadly interest both the extinction learning and cerebellar research communities. As such, this is a very timely and impactful manuscript. However, there are several points that could be addressed during the review process to strengthen the claims and enhance their value for readers and the broader scientific community.

(1) Reward Interpretation and Skin Conductance Responses (SCR)

A central premise of the manuscript is that 'unexpected omissions of expected aversive events' are rewarding, which plays a critical role in extinction learning. The authors also suggest that the cerebellum is involved in reward processing. However, it is unclear how this conclusion can be directly drawn from their task, which does not explicitly model 'reward.' Instead, the interpretation relies on SCR, which seems more indicative of association or prediction rather than reward per se. Is SCR a valid metric of reward experienced during the extinction of feared associations? Or could these findings reflect processes tied more closely to predictive learning? Please, discuss.

We thank the reviewer for raising this important point. We agree that skin conductance responses (SCRs) do not directly index reward. More generally, SCRs reflect autonomic arousal in response to salient or motivationally significant stimuli and are closely linked to expectancy and contingency awareness. In our study, SCRs served as a read-out of the participants’ expectation of a US, and were used to fit the hyperparameters of a reinforcement-learning-based deep learning model, which then provided per-trial estimates of prediction and prediction error values. These estimates capture predictive learning about the occurrence of the aversive US, rather than reward per se. The interpretation of unexpected US omissions as “reward-like” prediction errors relies on prior literature, particularly rodent studies showing that dopaminergic neurons in the VTA respond to omitted aversive stimuli and drive extinction learning via projections to the nucleus accumbens (Kalisch et al., 2019; Salinas-Hernández et al., 2018, 2023). We therefore interpret our cerebellar activations during unexpected omissions as being compatible with the processing of reward-like prediction errors, while acknowledging that this inference is indirect.

To clarify this reasoning, we made revisions to the Introduction and Discussion to (i) state explicitly that SCRs do not directly measure reward but were incorporated into the reinforcement learning model as an index of autonomic arousal related to US expectancy and predictive learning, and (ii) consistently replace the term “reward prediction error” with “reward-like prediction error” throughout.

(2) Reinforcement Agent and SCR Modeling

The modeling approach with the deep reinforcement agent treats SCR as a personalized expectation of shock for a given trial. However, this interpretation seems misaligned with participants' actual experience - they are aware of the shock but exhibit evolving responses to it over time. Why is this operationalization useful or valid? It would benefit the manuscript to provide a clearer justification for this approach.

This point is well taken. We did not collect trial-by-trial expectancy ratings, as frequent button-box responses would have induced cerebellar activations unrelated to fear (extinction) learning. Subjective expectancy was assessed only at the end of each experimental phase. As frequently done in the human fear conditioning literature, we used trial-by-trial SCR data (Lonsdorf et al., 2017). Although SCRs show correspondence with US expectancy ratings, they are inherently noisy and show substantial variability across trials and participants (Constantinou et al., 2021). Therefore, individual trial-by-trial responses cannot be used to directly infer US predictions. Accordingly, we used group-averaged SCR data to fit model hyperparameters in a grid search across parameter settings. The best-fitting hyperparameters were then applied to 100 randomly initialized agents, and their outputs were averaged to generate trial-wise estimates of predictions and prediction errors. These averaged values were used as parametric modulators in the fMRI analyses. We have revised the Introduction and Methods to make this procedure clearer.

(3) Clarity and Visualization of Results

The results section is challenging to follow, and the visualization and quantification of findings could be significantly improved. Terms like 'trending' appear frequently - what does this mean, and is it worth reporting? Adding clear statistical quantifications alongside additional visualizations (e.g., bar or violin plots of group means within specific subregions within the cerebellum, or grouped mean activity in VTA and DCN) would enhance clarity and allow readers to better assess the distribution and systematicity of effects. Furthermore, the figures are overly complex and difficult to read due to the heavy use of abbreviations. Consider splitting figures by either phase of the experiment or regions, and move some details to the supplemental material for improved readability.

We agree with the reviewer that the clarity of results can be improved and have revised the manuscript accordingly. Specifically:

(1) We use “trend-level” to refer to uncorrected voxelwise t-maps at p < 0.05, and “significant” to refer to TFCE/FWE-corrected effects at p < 0.05. This distinction was not sufficiently clear in the original figures. To address this, uncorrected t-maps are now displayed with a grey striped background frame, and colorbar labels have been enlarged to emphasize whether TFCE/FWE-corrected or uncorrected t-values are shown.

(2) We added a supplementary table (Table S7) reporting group-level summary statistics for all fMRI contrasts presented in the manuscript, including group means, standard deviations, effect sizes (Cohen’s d), and 95% confidence intervals for cerebellar cortex, cerebellar nuclei, and VTA VOIs. We hope that this helps with the interpretation of effect magnitude and variability across fMRI analyses.

(3) To improve readability, we split overly complex figures: Figure 2 now separates CS-related prediction from US-related presentation contrasts (which are now revised Figures 4 and 5), and Figure 3 separates event-based and parametric modulation contrasts (which are now revised Figures 6 and 7).

(4) We also reduced abbreviations in the figures, and provide full definitions and explanations also including the original abbreviations in the main text and figure captions for clarity.

We considered the suggestion to split figures further by region or by phase. However, we believe it is more informative to present the cerebellar cortex, nuclei, and VTA together for each contrast, and to keep all phases side by side, as this allows readers to directly assess commonalities across phases. We therefore chose to keep the same overall structure, but simplified the figures in other ways (e.g. splitting by contrast type) to improve overall readability. We hope that these changes address the reviewer’s concerns by simplifying the presentation, removing abbreviations, and providing clearer quantification of results.

(4) Theoretical Context for Paradigm Phases

The manuscript benefits from the comprehensive experimental paradigm, which includes multiple phases (acquisition, extinction, recall, reacquisition, re-extinction). This design has great potential for providing a more holistic view of conditioned fear learning and extinction. However, the manuscript lacks clarity on what insights can be drawn from these distinct phases. What theoretical framework underpins the different stages, and how should the results be interpreted in this context? At present, the findings seem like a display of similar patterns across phases without sufficient interpretation. Providing a stronger theoretical rationale and reorganizing the results by experimental phase could significantly improve readability and impact.

We thank the reviewer for this constructive suggestion. We would first like to mention that the primary aim of this manuscript is not to analyze differences between phases, but rather to highlight the commonalities. Across different learning contexts, we consistently observed reward-like prediction error-related activations in the cerebellum and VTA. This consistency and connectivity between the cerebellum and VTA, despite phase-to-phase differences, is the most important finding of our study.

We agree, however, that the manuscript did not sufficiently explain how each phase differs conceptually, which is important for readers to understand why the consistency of responses is notable. We therefore expanded the Introduction and Discussion to provide clearer theoretical context for each phase. More specifically, the phases can be understood as follows:

Extinction (day 2): Because acquisition was conducted with a 100% reinforcement rate, unexpected US omissions during initial extinction trials maximize reward-like prediction errors and yield stronger, more uniform expectations across participants compared to a partial reinforcement rate. This phase should therefore provide the clearest opportunity to observe cerebellar-VTA contributions to the processing of reward-like prediction errors.

Recall (day 3): Despite allowing for the consolidation of extinction learning, the recall test often still elicits conditioned fear responses to the CS+, that is, shows spontaneous recovery of the initial fear association (Bouton, 2002). In these trials, the non-occurrence of the US is unexpected. In this context, US omission-related activations reflect reward-like prediction errors during renewed fear responding in the presence of both a fear memory and an extinction memory. This contrasts with extinction training on day 2, where prediction errors arose primarily against the background of the recently acquired fear memory, without a competing extinction memory.

Reacquisition (day 3): Unlike acquisition, reacquisition used a partial reinforcement rate, such that non-reinforced CS+ trials were interspersed between reinforced CS+ trials (similar to the partially reinforced phase used by Ernst et al., 2019). Because reacquisition occurs in the presence of savings, that is, the presence of a previously acquired fear memory, US expectancy increases rapidly following reinforced trials and relearning occurs faster (Bouton, 2004). Importantly, partial reinforcement maintains high US expectancy and therefore allows prediction errors to remain sustained across omission trials (Figure 9).

Reextinction (day 3): Reextinction is an additional extinction phase but without a consolidation interval, and with an already established fear extinction memory. Because reextinction followed the partially reinforced reacquisition phase, prediction errors during early reextinction decayed more slowly than during extinction on day 2 (following the fully reinforced acquisition phase on day 1) (Figure 9). Together, reacquisition and reextinction were designed to maximize the number and persistence of unexpected US omissions, thereby providing additional opportunities to examine reward-like prediction-error signaling.

By clarifying this framework, we aim to show that while the learning context and history differ across phases, the consistent cerebellum-VTA activation and connectivity related to unexpected US omissions underlines the robustness of the effect. We chose not to reorganize the Results by phase, as our central conclusion rests on similarities rather than differences. Instead, we have clarified the theoretical background in the revised manuscript to help readers interpret both the commonalities and the potential sources of variability.

(5) Cerebellum-VTA Connectivity Analysis

The authors argue that the cerebellum modulates VTA activity, yet they perform the PPI analysis in the reverse direction. Why does this make sense? In their DCM analysis, they found a bidirectional relationship (both cerebellum - VTA and VTA-cerebellum), yet the discussion focused on connectivity from the cerebellum to VTA. A more careful interpretation of the connectivity findings would be useful - especially the strong claims in the discussion on the cerebellum providing the reward signal to the VTA should be tempered.

We thank the reviewer for highlighting this issue. In our primary analysis, we used the VTA as the PPI seed and observed trend-level connectivity with the cerebellum. When we reversed the analysis and used the cerebellar volume of interest (VOI) from the conjunction analysis as the seed, effects in the VTA were substantially weaker. We believe this reflects the broad connectivity profile of the cerebellar VOI (i.e., not specific to the VTA) as well as general limitations of PPI in our study, including the small number of unexpected omission trials and the lack of specificity to reward-like prediction errors (e.g., connectivity also appeared during US presentation). For transparency, we now report the cerebellar-seed PPI results in the Supplementary information (Figure S3). Given their limited robustness, we chose not to include the corresponding VTA maps in the main figures.

Finally, we agree that our conclusions regarding cerebellum-VTA interactions should be framed more cautiously. While the DCM analyses support bidirectional connectivity, our original discussion placed disproportionate emphasis on cerebellum-to-VTA influences. We have revised the text to provide a more balanced interpretation that also considers VTA-to-cerebellum connectivity.

**Reviewer #2 (Public review):**

Summary

Building upon the group's previous work, this study used a 3-day threat acquisition, extinction, recall, reextinction, and reacquisition paradigm with 7T imaging to probe the mechanism by which the cerebellum contributes to fear extinction learning. The authors hypothesize this may be via its connection to the VTA, a known modulator of fear extinction due to its role in reward processing. Using complementary analysis methods, the authors demonstrate that activity with the cerebellum, DNC, and VTA is modulated by predictions about the occurrence of the US, which shows regional specificity. They show trend-level evidence that there is increased functional connectivity between the cerebellum and VTA during all phases of the paradigm with unexpected omissions. They also present a DCM which indicates that the cerebellum could positively modulate VTA activity during extinction learning. This study adds to a growing literature supporting the role of the historically overlooked cerebellum in the control of emotions and suggests that an interaction between the cerebellum and VTA should be considered in the existing model of the fear extinction network.

Strengths

The authors address their research question using a number of complementary methods, including parametric modulation by model-derived expectation parameters, PPI, and DCM, in a logical and easily understood way. I feel the authors provide a balanced interpretation of their findings, presenting numerous interpretations and offering insight with regard to reward vs attention or unsigned prediction errors and the directionality of the interaction they identify. The manuscript is a timely addition to growing literature highlighting the role of the cerebellum in fear conditioning, and emotion generation and regulation more generally.

Weaknesses

Subjective and skin conductance responses do not completely support the success of the learning paradigm. For example, CS+/CS- differentiation in both domains persisted after extinction training. I do not feel that this negates the findings of this manuscript, though it raises questions about the parametric modulators used, and the interpretation of the neural mechanisms proposed if they do not strongly relate to updated subjective appraisals (the goal of extinction therapy). My interpretation of the manuscript suggests there are some key results based upon contrasts that have as few as three events; I am a little unsure about the power and reliability of these effects, though I await author clarification on this matter. There are a number of unaddressed deviations from the pre-registered protocol that I have asked the authors to elaborate upon.

We thank the reviewer for the thoughtful and constructive evaluation of our work. We appreciate that the manuscript and methods were found to be clearly presented, and we welcome the suggestions for clarification and improvement. Below we address the specific concerns regarding extinction learning in behavioral measures, the reliability of event-based contrasts with few trials, and deviations from the preregistration.

Extinction in self-reports and skin conductance responses (SCRs)

The reviewer is correct that CS+/CS- differentiation persisted after extinction. Although there was no differentiation in SCRs at the end of extinction, post-extinction self-reports continued to do so, albeit to a lesser degree, which is in line with previous literature on dissociation of outcome measures during fear conditioning (Lipp et al., 2003). This residual subjective differentiation is also consistent with extinction forming an inhibitory memory trace that suppresses, rather than erases, the original fear association (Bouton, 2002; Milad & Quirk, 2012), and a single extinction session is often insufficient to eliminate differential responding (Craske et al., 2014; Vervliet et al., 2013). However, both measures showed significant effects of extinction learning.

We included additional analyses of self-reports across phases. Importantly, CS+ ratings were significantly reduced during extinction and recall compared to acquisition (all p ≤ 0.001), whereas CS- ratings remained unchanged (all p > 0.532). This pattern demonstrates that the magnitude of the CS+/CS- difference was significantly reduced relative to acquisition, indicating that extinction learning did occur (Doubliez et al., 2025).

For physiological responses, extinction learning was shown in PSRs but not conclusively in SCRs. PSRs showed a significant reduction of CS+ responses across extinction, while CS- responses remained unchanged. SCRs showed a reduction of CS+/CS- differentiation across extinction; however, this effect remained at trend level, as the Stimulus x Time interaction did not reach significance (p = 0.053). This pattern is consistent with early differentiation followed by rapid attenuation under the full reinforcement structure of the paradigm (100% reinforcement during acquisition and 0% during extinction). Under such conditions, participants rapidly learn that the US is no longer delivered during extinction, such that physiological responses are largely confined to the first few trials, leaving limited power to detect extinction effects in noisier measures such as SCRs. To address the lower robustness of SCR effects, as recommended by the reviewer, we therefore included PSRs in the main Results section, which provide converging physiological evidence for extinction learning.

Of note, on day 3, both physiological measures and self-reports again showed CS+/CS- differentiation, consistent with spontaneous recovery, a well-established phenomenon reflecting the persistence of the original fear trace after consolidation (Bouton, 2002; Vervliet et al., 2013).

Taken together, these findings demonstrate that the paradigm successfully induced both acquisition and extinction of conditioned fear, even though residual fear responses persisted.

Reliability of event-based contrasts with three trials

The initial decision to use three events for event-based contrasts was based on SCR and PSR data, which showed that differentiation between CS+ and CS- occurred almost exclusively in the first few trials of extinction and recall. Consistent with the full reinforcement described above, prediction errors were expected to be high in the very first extinction trials, and to decay rapidly. Thus, the usual half-block division (e.g., first eight trials) would have included many trials without meaningful prediction errors.

We acknowledge that contrasts based on three trials provide limited statistical power. To address this concern, we added a supplementary table showing summary statistics for contrast estimates in the cerebellar cortex, cerebellar nuclei, and VTA VOIs across all fMRI analyses (Table S7), including both the event-based and parametric modulation approaches. Importantly, the event-based contrasts showed moderate to strong effects despite being restricted to the first three unexpected omission trials. Moreover, the parametric modulation analyses, which incorporate all available trials, yielded results that were consistent with the three-trial event-based contrasts and with the patterns shown in the main figures. This convergence between event-based and parametric approaches strengthens our confidence that the observed effects are reliable.

Deviations from preregistration

We acknowledge that deviations from the preregistered protocol were not fully documented and have now added this information. The main deviation concerned our event-based analyses: while the preregistration planned early vs. late block comparisons, in practice the rapid decay of SCRs under our 100% and 0% reinforcement rates rendered later trials uninformative for prediction error analyses. We therefore focused on the first three trials, when prediction errors are expected to be present. These behavioral findings are also consistent with Doubliez et al. (2025), who used the same paradigm and observed similar rapid SCR decay. Other deviations, such as not reporting exploratory whole-brain DCM analyses, are now clearly stated for transparency.

**Recommendations for the authors:**

**Reviewer #1 (Recommendations for the authors):**

Minor Point - Paradigm Details

Providing additional details about the experimental paradigm in the main text (e.g., the nature of the visual stimuli associated with shocks) would enhance the manuscript's clarity. Some of the information currently in supplementary Figure 5 could be incorporated into the main text to enhance the understanding of the paradigm

We agree that the current structure reduces clarity, as the paradigm is only explained in detail after the results. To improve readability, we have moved parts of Figure 5 (illustrating the paradigm and scanner setup) to the beginning of the manuscript (now revised Figure 1). In addition, information from Figure 5, including details of the visual stimuli, is now added to the Introduction.

**Reviewer #2 (Recommendations for the authors):**

Methods

Can the authors please clarify what part of the task went into [US post CS+ > no US post CS-] contrast? Is this the time immediately after the CS presentations, when the US has just occurred/not occurred, or rather more like the CS+>CS- contrast except including trials confounded by the US (i.e. [CS+/US > CS -])?

The contrasts are based on an event-related separation of CS and US. The CS was presented for 6 seconds, with its onset modeled in the GLM as a zero-duration event (delta function). The CS offset coincided with either the delivery or omission of the US, which was likewise modeled as a zero-duration event. Thus, CS onset and offset were modeled separately. The no-US events were further distinguished by whether they followed a CS+ or a CS-. Accordingly, we analyzed both CS and US-related contrasts; for example, the CS+ > CS- contrast reflects CS-related differentiation at CS onset (0 s), whereas [US post CS+ > no US post CS-] reflects (no-)US-related activity at CS offset (6 s; US delivered from 5.9-6.0 s). We have added further clarification to the Methods section.

I was a bit unclear on what this sentence of the methods meant "Notably, all single trials comprised CS+ trials, with CS- trials also being modeled as single trials to facilitate paired analysis", does this mean that some contrasts had 6 events in total - e.g. the first 3 unexpected omissions vs 3 x CS-. If so, which CS- were selected for the comparison?

We agree that this sentence was unclear and have revised it. Our intention was to describe that when CS+ trials were modeled as single trials in the GLM (e.g., each CS+ onset and its associated [no-]US event modeled as separate regressors), the CS- trials were modeled in the same way. This ensured that paired analyses would be possible if required.

For reacquisition and reextinction, single-trial modeling was necessary, as the last unexpected omission of reacquisition is also the first unexpected omission of reextinction. Modeling trials separately allows us to examine the first three unexpected US omissions in each phase independently.

The event-based contrasts for unexpected US omissions were defined in line with a previous study of our group. For example, during extinction we contrasted the first three unexpected US omissions following CS+ with all expected omissions following CS- (i.e. [first 3 no US post CS+ > no US post CS-], corresponding to 3 vs. 16 events). The weights of events were automatically scaled by SPM12 so that both sides of the contrast carried equal total weight (e.g. positive events weighted 1/3, negative events weighted -1/16). This procedure matches the approach in Ernst et al. (2019), where in partially reinforced acquisition 6 unexpected omissions after CS+ were contrasted with 16 expected omissions after CS-.

More generally, can the authors please comment on the power and reliability of analyses that include only 3 events in a condition [e.g. the first 3 unexpected omissions]?

It is not clear if the (US post CS+ > no US post CS-) phases were included. In your pre-registration you say "we will use a "no US post CS+ > no US post CS-" fMRI contrast, where "no US post CS+" designates unexpected omission events in early extinction, early recall (depending on behavioral data which might indicate a return of fear) and a volatile phase (where unexpected omissions occur in the first part of the volatile phase, i.e. reacquisition).", but my reading of the manuscript was that it included both early and late "see 1st level analysis = US post CS+, no US post CS+, no US post CS- separately for each phase; 2nd level = contrast included unexpected omission of the US (no US post CS+ > no US post CS-)". Please clarify and if necessary explain the deviation from preregistration.

We agree that this point requires clarification. In the preregistration, we planned to divide phases into early and late blocks (no US post CS+ > no US post CS-). However, as already outlined in our response (Reviewer 2, public review response: Reliability of event-based contrasts with three trials), both our preliminary behavioral data and subsequent modeling analyses indicated that differentiation between CS+ and CS- declined extremely rapidly under the 100% reinforcement schedule, leaving likely little or no prediction error beyond the first few trials. Based on this, we adapted the event-based analyses to focus on the first three unexpected omission trials in extinction, recall, and reextinction, where prediction errors are expected to be present. In reacquisition, only three omission events occur by design (83% reinforcement), so this naturally constrained the analysis to three trials. We now explicitly describe this deviation from the preregistration in the revised manuscript.

As outlined in the same response, we recognize that contrasts based on three trials provide limited statistical power, and addressed this point by providing additional summary VOI statistics of contrast estimates for both event-based and parametric modulation contrasts, which show moderate-to-strong effect sizes and convergence across methods, which we argue supports that using the first three trials is a reliable approach (Reviewer 1, public review response, point (3) Clarity and Visualization of Results).

Finally, with regard to the reviewer’s specific question: yes, US post CS+ > no US post CS- contrasts were examined for acquisition training, primarily to demonstrate US-related activation (see revised Figure 3).

Results

Page 5 + 6: Including the interaction effects for pupil size responses during extinction and reextinction in the SCR section seems unjustified. I appreciate that the SCR data does not significantly support the key claim that extinction learning towards the CS+ occurred, but I do not feel it is acceptable to draw from the other measure for this effect alone. If the PSR measure is of primary/significant importance to support the validity of your paradigm, please consider adding all of these results to the main manuscript.

We agree with this point and have moved the PSR analysis to the main manuscript. In addition, the SCR Results section no longer includes the PSR analyses, and clearly states the absence of a significant Stimulus x Time interaction effect in extinction (p = 0.053). For completeness, we additionally report trend-level post hoc tests showing CS+/CS- differentiation during early extinction but not during late extinction, consistent with an initial differentiation that attenuates across extinction training.

Subjective and (some) skin conductance responses do not completely support the success of the learning paradigm. For example, CS+/CS- differentiation in both subjective domains and SCRs persisted after extinction training. Can the authors comment on how this might influence the interpretation of their results more generally? What does it mean if these expectations do not appropriately translate to updated subjective appraisals in your participants, contrary to the model from which the parametric modulators were derived would predict?

The persistence of CS+/CS- differentiation in self-reports after extinction, and the return of CS+/CS- differentiation in both self-reports and physiological measures during the recall test, is not unexpected. For self-reports administered after extinction, such persistent CS+/CS- differences are commonly observed in the human fear extinction literature (Hermans et al., 2006; see also Lipp et al., 2003), and may reflect that initial extinction learning establishes a new inhibitory association that suppresses, but does not erase, the original fear memory (Bouton, 2002). At recall on day 3, the remaining differentiation in both self-reports and physiological responses is consistent with spontaneous recovery, a well-documented phenomenon in extinction research (Bouton, 2002). As noted earlier (Reviewer 2, public review response: Extinction in self-reports and skin conductance responses (SCRs)), additional analyses showed that ratings were significantly reduced after extinction and recall compared to acquisition. Thus, while residual differentiation in self-reports remained after extinction and recall, its magnitude was diminished, indicating that extinction learning occurred but was incomplete. This pattern is consistent with partial updating of subjective appraisals in accordance with the reinforcement-learning model used to derive the parametric modulators, rather than a failure of updating.

Figures

Figure 1: Please ensure that the summary of your results in the figure legend is consistent with the quantitative results reported. Example 1: "On day 2, there was a loss of differentiation during extinction training.", however, a significant effect of the stimulus, and time remained (but no interaction). Please tone down this interpretation, or make it clearer how the difference in the initial extinction trials was quantified. If the ANOVA-type analysis was only performed in the first half, this was not clear. Example 2: "During initial reacquisition, there were again differential responses to the CS+ and CS-, which decreased in reextinction and the unexpected US phase". I appreciate that you refer to the difference decreasing, rather than disappearing altogether, but the magnitude of this difference is not reported in the manuscript, and there does remain a significant difference in the amplitude.

We thank the reviewer for this helpful feedback. We have revised the figure legends to tone down overly strong statements and ensure that all descriptions are in correspondence with the quantitative results. For clarity, we have also added significance markers for (trend-level) post hoc comparisons (CS+/CS- differentiation within early and late blocks for each phase) to revised Figures 2 and 3 displaying SCRs and PSRs.

Figure 2, 3, 4: I found it quite confusing to have uncorrected and corrected results displayed in the same way in the same figure. E.g. Figure 2A which, as far as I can tell shows trend-level results for the cerebellum, and corrected results for the VTA. For Figures 2 and 3 it was also not immediately clear which colour bar related to which map. Figure 4A appeared to be missing colour bars. I suggest the authors consider (as much as possible) standardising the colour bar scales, such that the maps across figures/sub-plots are more directly comparable, and differentiate more clearly between corrected and uncorrected results. The 3D renders in Figures 2 and 3 are a little hard to see - would it be possible to make it not so transparent?

We use “trend-level” to refer to uncorrected voxelwise t-maps at p < 0.05, and “significant” to refer to TFCE/FWE-corrected effects at p < 0.05. This distinction was not sufficiently clear in the original figures. In the revised figures, uncorrected t-maps are displayed with a grey striped background frame. Colorbar scales were not standardized, as different panels display different statistical quantities (TFCE values versus t-values), and scaling was chosen to visualize variation within each contrast rather than enforce comparability across panels, which would have reduced interpretability. In addition, the missing colorbar in Figure 8A (formerly Figure 4A) has now been added; it matches the colorbar shown in Figure 8B. See also Reviewer 1, public review response, point (3) Clarity and Visualization of Results.

Is it possible to annotate significant effects on Figure 1 and Supplement Figure 1? The use of square markers makes it quite hard to tell the value of each point, which, given the small scale of the y-axis is quite important for interpretation. Could the authors consider remaking these plots with smaller dots?

We have added post hoc significance markers to Figures 2 and 3 displaying SCRs and PSRs to facilitate interpretation. These markers reflect post hoc comparisons of CS+/CS- differentiation within early and late blocks. In cases where the Stimulus x Time interaction was not significant, the corresponding post hoc markers are still shown but are indicated in red to denote their trend-level status. In addition, the plots have been remade with smaller dots to make individual values clearer.

Discussion

The authors state "Because aversive stimulus presentation results in pronounced cerebellar activations, we were unable to separate cerebellar activation related to the unexpected (initial acquisition trials) and the expected (late acquisition trials) presentation of the US." Could the authors compare between early[CS+>CS-] and late[CS+>CS-] acquisition (which I believe were created in the event-based analysis but results not reported), or between the first 3[CS+ with US>CS-] and later [CS+ with US>CS-] to assess this?

In our terminology, the suggested comparisons (early vs. late [CS+ > CS-] or first three vs. last three [CS+ > CS-]) reflect changes in US prediction rather than prediction error. The statement in the Discussion refers specifically to cerebellar activation during US presentation, where distinguishing between expected and unexpected presentations is complicated by the strong cerebellar activation elicited by the electrical US itself. Moreover, when comparing early “unexpected” US presentations with later “expected” ones, the relatively higher activity in early trials could reflect habituation of the US sensation (i.e., non-associative learning) rather than a prediction error, making interpretation difficult.

Because the current manuscript focuses on reward-like prediction errors, we did not report these US prediction or presentation contrasts in detail. In brief, the suggested comparisons of early versus late CS-related differentiation (CS+ > CS-), revealed only limited trend-level activity. In contrast, US-related responses during acquisition showed robust activations in the cerebellar cortex, DCN, and VTA across the acquisition phase. Comparisons between the first three US presentations and later US presentations showed broadly distributed and stronger responses during early acquisition than during later US presentations. This pattern seems to be more consistent with non-associative effects, such as sensory habituation to the electrical stimulation, rather than with prediction-error–related processing. We have therefore not included them in the manuscript, but would be open to providing them in the Supplementary Information if the editor or reviewers consider them essential.

General

In your pre-registered analysis plan you state "we will explore the use of DCM in a larger network that encompasses known constituents of the fear extinction network, in addition to the cerebellum and VTA.". You have plenty of results to discuss in the current manuscript and adding this may complicate the narrative, but that being said, please either perform and include this analysis as you proposed or explicitly mention why this was not completed. You could also consider adding a whole-brain activation map for the key phases of the experiment. Please also double-check other pre-registered points, for example - the sample size justification is also different.

We decided not to include whole-brain DCM analyses in this manuscript and not to report whole-brain activation results extensively, as the study was primarily hypothesis-driven with a focus on cerebellum-VTA interactions. While we recognize that whole-brain analyses are of interest and plan to explore them in future work, they were considered outside the scope of the current paper. This deviation from the preregistration is now explicitly noted in the revised manuscript.

Regarding the sample size justification, the preregistration contained an error: the parameters were reported incorrectly. The correct sample size justification was already provided in the original 2019 grant application and is correctly reported in the current manuscript. The underlying power analysis was the same, but with different alpha levels depending on whether the study involved healthy participants (where larger samples are feasible) or rare patient populations (where stricter alpha levels are not practical). We have clarified this point in the manuscript under deviations from the preregistration.

Additional changes made in manuscript by authors

To provide a complete overview, we also note changes made independently of specific reviewer comments:

Methods

In the computational modeling section, “reextinction” was mistakenly mentioned where “reacquisition phase” was intended (the initial phase of the volatile phase before experience replay). This has been corrected.

The term “trial sequence” is used in computational modeling, whereas counterbalancing in the fear conditioning methods used different terminology. We added a clarifying sentence in the modeling section to make this consistent.

References in the pupil size analysis section (Jentsch et al. 2020; Mathôt et al. 2017) were misplaced and have now been moved earlier in the sentence.

The citation for MRIcroGL software was updated to the current Nature Methods reference.

We added a reference to Doubliez et al. 2025 which used the same three-day paradigm in a behavioral study showing similar physiological responses.

Supplementary information

During revision, we noted that the SCR statistics had been computed on an earlier preprocessed dataset version, whereas the finalized corrected dataset was already used for plotting and for estimating prediction and prediction-error values in the reinforcement-learning model. We therefore recomputed the SCR statistics on the finalized dataset for the sake of consistency; this did not change any main effects, interactions, or conclusions, with the only difference being an exploratory late-acquisition CS+/CS- post hoc shifting from non-significant to p < 0.05 (interaction still non-significant). Updated statistics are reported in the Supplementary information.

Post hoc significant differences in Table S3 are now marked in bold, as the formatting was missing previously.

To align behavioral analyses more closely with the event-based fMRI approach, we additionally examined physiological responses using a first three versus last three trial division within each phase. These analyses yielded patterns consistent with those obtained using the original early/late block division and are reported in the Supplementary Information.

We added a new supplementary figure (Figure S4) showing the location of the cerebellar VOI on a SUIT flatmap and added a corresponding cross-reference in the Methods section (Volumes of interest (VOI) definition)

References

Bouton, M. E. (2002). Context, ambiguity, and unlearning: sources of relapse after behavioral extinction. Biological Psychiatry, 52(10), 976–986. https://doi.org/10.1016/S0006-3223(02)01546-9

Bouton, M. E. (2004). Context and Behavioral Processes in Extinction: Table 1. Learning & Memory, 11(5), 485–494. https://doi.org/10.1101/lm.78804

Constantinou, E., Purves, K. L., McGregor, T., Lester, K. J., Barry, T. J., Treanor, M., Craske, M. G., & Eley, T. C. (2021). Measuring fear: Association among different measures of fear learning. Journal of Behavior Therapy and Experimental Psychiatry, 70(September 2020), 101618. https://doi.org/10.1016/j.jbtep.2020.101618

Craske, M. G., Treanor, M., Conway, C. C., Zbozinek, T., & Vervliet, B. (2014). Maximizing exposure therapy: An inhibitory learning approach. Behaviour Research and Therapy, 58, 10–23. https://doi.org/10.1016/j.brat.2014.04.006

Doubliez, A., Köster, K., Müntefering, L., Nio, E., Diekmann, N., Thieme, A., Albayrak, B., Nicksirat, S. A., Erdlenbruch, F., Batsikadze, G., Ernst, T. M., Cheng, S., Merz, C. J., & Timmann, D. (2025). Dopaminergic drugs modulate fear extinction-related processes in humans, but effects are mild. Brain Communications, 7(5), fcaf333. https://doi.org/10.1093/braincomms/fcaf333

Ernst, T. M., Brol, A. E., Gratz, M., Ritter, C., Bingel, U., Schlamann, M., Maderwald, S., Quick, H. H., Merz, C. J., & Timmann, D. (2019). The cerebellum is involved in processing of predictions and prediction errors in a fear conditioning paradigm. ELife, 8, e46831. https://doi.org/10.7554/eLife.46831

Hermans, D., Craske, M. G., Mineka, S., & Lovibond, P. F. (2006). Extinction in Human Fear Conditioning. Biological Psychiatry, 60(4), 361–368. https://doi.org/10.1016/j.biopsych.2005.10.006

Kalisch, R., Gerlicher, A. M. V., & Duvarci, S. (2019). A Dopaminergic Basis for Fear Extinction. Trends in Cognitive Sciences, 23(4), 274–277. https://doi.org/10.1016/j.tics.2019.01.013

Lipp, O. V., Oughton, N., & LeLievre, J. (2003). Evaluative learning in human Pavlovian conditioning: Extinct, but still there? Learning and Motivation, 34(3), 219–239. https://doi.org/10.1016/S0023-9690(03)00011-0

Lonsdorf, T. B., Menz, M. M., Andreatta, M., Fullana, M. A., Golkar, A., Haaker, J., Heitland, I., Hermann, A., Kuhn, M., Kruse, O., Meir Drexler, S., Meulders, A., Nees, F., Pittig, A., Richter, J., Römer, S., Shiban, Y., Schmitz, A., Straube, B., … Merz, C. J. (2017). Don’t fear ‘fear conditioning’: Methodological considerations for the design and analysis of studies on human fear acquisition, extinction, and return of fear. Neuroscience and Biobehavioral Reviews, 77, 247–285. https://doi.org/10.1016/j.neubiorev.2017.02.026

Milad, M. R., & Quirk, G. J. (2012). Fear Extinction as a Model for Translational Neuroscience: Ten Years of Progress. Annual Review of Psychology, 63(1), 129–151. https://doi.org/10.1146/annurev.psych.121208.131631

Salinas-Hernández, X. I., Vogel, P., Betz, S., Kalisch, R., Sigurdsson, T., & Duvarci, S. (2018). Dopamine neurons drive fear extinction learning by signaling the omission of expected aversive outcomes. ELife, 7, e38818. https://doi.org/10.7554/eLife.38818

Salinas-Hernández, X. I., Zafiri, D., Sigurdsson, T., & Duvarci, S. (2023). Functional architecture of dopamine neurons driving fear extinction learning. Neuron, 111(23), 3854-3870.e5. https://doi.org/10.1016/j.neuron.2023.08.025

Vervliet, B., Craske, M. G., & Hermans, D. (2013). Fear extinction and relapse: State of the art. Annual Review of Clinical Psychology, 9(March 2013), 215–248. https://doi.org/10.1146/annurev-clinpsy-050212-185542
